# Investigating the mechanisms of Modified Xiaoyaosan (tiaogan-liqi prescription) in suppressing the progression of atherosclerosis, by means of integrative pharmacology and experimental validation

**DOI:** 10.18632/aging.202832

**Published:** 2021-04-04

**Authors:** Mingtai Chen, Yong Luo, Ling Men, Bo Lin, Haidan Lin, Ying Li, Guofu Zhong, Xiaoling Zhong, Wenjun Fu, Hua Zhou, Guangdong Tong, Qiang Liu, Jienan Luan

**Affiliations:** 1 Department of Cardiovascular Disease, Shenzhen Traditional Chinese Medicine Hospital, Shenzhen, China; 2 Faculty of Chinese Medicine and State Key Laboratory of Quality Research in Chinese Medicine, Macau University of Science and Technology, Macao, China; 3 Centre for Integrative Medicine, School of Basic Medical Science, Guangzhou University of Chinese Medicine, Guangzhou, Guangdong, China; 4 Nephrology Department, Shenzhen Traditional Chinese Medicine Hospital, Shenzhen, China; 5 Intensive Care Unit, Shenzhen Traditional Chinese Medicine Hospital, Shenzhen, China; 6 Reproductive Health Department, Shenzhen Traditional Chinese Medicine Hospital, Shenzhen, China; 7 Department of Liver Disease, Shenzhen Traditional Chinese Medicine Hospital, Guangzhou University of Chinese Medicine, Shenzhen, China

**Keywords:** atherosclerosis, traditional Chinese medicine, Modified Xiaoyaosan prescription, integrative pharmacology, network analysis

## Abstract

Atherosclerosis (AS)-related diseases remain among the leading causes of death worldwide. Modified Xiaoyaosan (also called Tiaogan-Liqi prescription, TGLQ), a traditional Chinese medical formulation, has been widely applied in the treatment of AS-related diseases. The aim of this study was to investigate the underlying pharmacological mechanisms of TGLQ in acting on AS. A total of 548 chemical compounds contained in TGLQ, and 969 putative targets, were collected from the Computation Platform for Integrative Pharmacology of Traditional Chinese Medicine, while 1005 therapeutic targets for the treatment of AS were obtained from the DisGeNET, TTD and CTD databases. Moreover, the 63 key targets were screened by the intersection of the targets above, and by network topological analysis. Further functional enrichment analysis showed that the key targets were significantly associated with regulation of the immune system and inflammation, improvement of lipid and glucose metabolism, regulation of the neuroendocrine system and anti-thrombosis effect. The *in vivo* experiments confirmed that TGLQ could reduce plasma lipid profiles and plasma inflammatory cytokines, and also inhibit AS plaque formation, within the AS model ApoE-/- mice. The *in vitro* experiments validated the hypothesis that TGLQ could significantly reduce intracellular lipid accumulation, suppress the production of inflammatory cytokines of macrophages induced by oxidized-LDL, and inhibit the protein expression of heat shock protein 90 and toll-like receptor 4. This study identified a list of key targets of TGLQ in the treatment of AS by applying an integrative pharmacology approach, which was validated by *in vivo* and *in vitro* experimentation.

## INTRODUCTION

Acute cardiovascular and cerebrovascular events entail high levels of mortality and disability, which are strongly related to the rupture of atherosclerotic plaque [1]. Atherosclerosis (AS) is a dyslipidemia and chronic inflammatory disease, the complex mechanisms of which have not been clarified. Although several interventions of therapeutic strategies, such as modification of lifestyle factors, moderate exercise, statin treatment and antihypertensive drugs have proven beneficial for AS, the development of pathological processes in around 50% of AS patients was not impeded [2, 3]. Moreover, the high cost and unavoidable adverse effects of current medicine in the context of long-term treatment cannot be ignored. In addition to the therapeutic strategies above, traditional Chinese medicine (TCM) may provide an alternative and complementary therapy for AS [4, 5].

In TCM theory, the aetiology and pathogenesis of AS are summarized as “qi stagnation and blood stagnation”, which is closely associated with “liver dysfunction”. In line with the aetiology and pathogenesis cited above, we applied the TCM treatment principle of “improving the liver function”, “regulating qi stagnation” and “activating blood circulation” to the treatment of AS [6]. Modified Xiaoyaosan (also called Tiaogan-Liqi prescription, TGLQ) is based on Xiaoyaosan, a herb that has been used widely for treating AS-related diseases since 1151 AD, as being representative of “improving the liver function” and “regulating qi stagnation” prescriptions [7]. The TGLQ prescription is composed of ten herbs, including Radix Bupleuri (Chinese name: Chaihu, CH, roots of *Bupleurum Chinense DC.*), Chuanxiong Rhizoma (Chinese name: Chuanxiong, CX, rhizomes of *Ligusticum chuanxiong Hort.*), Curcumae Radix (Chinese name: Yujin, YJ, root tubers of *Curcuma wenyujin Y.H. Chen et C. Ling*), Angelicae Sinensis Radix (Chinese name: Danggui, DG, roots of *Angelica sinensis (Oliv.) Diels*), Paeoniae Radix Alba (Chinese name: Baishao, BS, roots of *Paeonia lactiflora Pall.*), Macrocephalae Rhizoma (Chinese name: Baizhu, BZ, rhizomes of *Atractylodes macrocephala Koidz.*), Poria (Chinese name: Fuling, FL, sclerotium of *Poriacocos (Schw.)Wolf*), Radix Rhizoma Glycyrrhizae (Chinese name: Gancao, GC, roots and rhizomes of *Glycyrrhiza uralensis Fisch.*), Herba Menthae (Chinese name: Bohe, BH, above-ground parts of *Mentha haplocalyx Briq.*) and Zingiberis Rhizoma Recens (Chinese name: Shengjiang, SJ, rhizomes of *Zingiber officinale Rosc.*).

Among the herbs cited above, CH contributes to regulating qi, which is one of the critical functional materials within the body, by soothing the liver [8]. Not only can CX regulate qi, but it also can activate blood circulation, which is another important functional element [9]. According to the Chinese-medicine compatibility theory of “sovereign, ministerial, adjuvant and messenger”, the ten herbs in TGLQ can be classified as sovereign, ministerial, adjuvant and messenger herbs. CH and CX are sovereign herbs in the TGLQ prescription, mainly targeting qi and “blood stagnation”. BS, DG and YJ play their respective roles as ministerial herbs, enhancing the effects of sovereign herbs. Moreover, BS and DG can either activate blood circulation or tonify blood [10]. YJ can also improve qi movement and remove blood stasis [11]. FL, BZ and GC act as adjuvant herbs, mainly fortifying the spleen, replenishing qi and “drying dampness” [10]. BH and SJ are messenger herbs, guiding herbs into the associated meridians. Furthermore, BH can either soothe the liver or “clear heat”; SJ can warm the middle of the body and resolve phlegm. Recently, increasing clinical evidence has implied that the prescription above is of benefit to AS patients by alleviating angina pectoris symptoms, lowering blood pressure, regulating blood lipids, and inhibiting inflammation [12, 13]. In addition, our previous study indicated that the prescription could inhibit the pathological process of AS in high-fat diet ApoE^-/-^ mice [14]. Nonetheless, it has been difficult to clarify completely the underlying molecular mechanisms of the TGLQ prescription in acting on AS via a conventional pharmacological approach, because there are complex and various components in the prescription.

Integrative pharmacology or network pharmacology, as an element of systems-biology approaches, provides an efficient and comprehensive approach to revealing the holistic pharmacological effects of TCM prescriptions at the molecular level [15–17]. The methodology shifts the TCM pharmacological research model from “one drug, one target” to “multi-component therapeutics, biological networks”, which accords with the holism and complexity of TCM characteristics. In this study, we aimed to investigate the underlying mechanisms of the TGLQ prescription in acting on AS, through the following steps: (1) We first collected the putative targets of each component in the TGLQ prescription from the Integrative Pharmacology-based Research Platform of Traditional Chinese Medicine (TCMIP, Version 2.0, http://www.tcmip.cn) and the therapeutic targets of AS from several databases; (2) We then extracted the common targets from the intersection of TGLQ-prescription putative targets and AS therapeutic targets; (3) To obtain the key targets, we screened them from the common targets through network topological analysis; (4) To investigate the underlying mechanisms of the TGLQ prescription acting on AS, we subjected the key targets above to functional enrichment analysis; (5) We validated the related targets from network analysis-based data by *in vivo* and *vitro* experiments [2].

## RESULTS

### Prediction of TGLQ prescription putative targets

A total of 969 putative targets and 548 chemical compounds contained in TGLQ were collected from TCMIP. Among these, there were 305 putative targets for CH, 332 for CX, 26 for YJ, 333 for DG, 420 for BS, 356 for BZ, 232 for FL, 288 for GC, 285 for BH and 247 for SJ. There were a large number of common putative targets for the ten herbs contained in TGLQ (Table 1), indicating the potential herb-herb interactions through these common targets. Detailed information regarding the TGLQ components and putative targets are provided in Supplementary Tables 1, 2.

**Table 1:** The number of common putative targets among ten herbs contained in TGLQ.

	***Bupleurum Chinense DC*.** **(Chaihu, CH) (305)**	***Ligusticum chuanxiong Hort*. (Chuanxiong, CX) (332)**	***Curcuma wenyujin Y.H.Chen et C.Ling* (Yujin, YJ) (26)**	***Angelica sinensis (Oliv.)Diels* (Danggui, DG) (333)**	***Paeonia lactiflora Pall*.** **(Baishao, BS) (420)**	***Atractylodes macrocephala Koidz*. (Baizhu, BZ) (356)**	***Poria cocos(Schw.) Wolf* (Fuling, FL) (232)**	***Glycyrrhiza uralensis Fisch*. (Gancao, GC) (288)**	***Mentha haplocalyx Briq*.** **(Bohe, BH) (285)**	***Zingiber officinale Rosc*. (Shengjiang, SJ) (247)**
*Bupleurum Chinense DC.* (Chaihu, CH) (305)	-	188	17	192	220	101	177	141	32	56
*Ligusticum chuanxiong Hort.* (Chuanxiong, CX) (332)	188	-	15	252	159	73	177	103	48	76
*Curcuma wenyujin Y.H.Chen et C.Ling* (Yujin, YJ) (26)	17	15	-	15	15	8	14	14	11	21
*Angelica sinensis (Oliv.)Diels* (Danggui, DG) (333)	192	252	15	-	151	81	170	84	35	81
*Paeonia lactiflora Pall.* ^(*Baishao, BS*)^ *(420)*	220	159	15	151	-	157	140	214	100	111
*Atractylodes macrocephala Koidz.* (Baizhu, BZ) (356)	101	73	8	81	157	-	66	69	191	181
*Poria cocos(Schw.)Wolf* (Fuling, FL) (232)	177	177	14	170	140	66	-	111	30	45
*Glycyrrhiza uralensis Fisch.* (Gancao, GC) (288)	141	103	14	84	214	69	111	-	39	42
*Mentha haplocalyx Briq.* ^(*Bohe, BH*)^ *(285)*	32	48	11	35	100	191	30	39	-	152
*Zingiber officinale Rosc.* (Shengjiang, SJ) (247)	56	76	21	81	111	181	45	42	152	-

### Overview of the TGLQ prescription

**Figure 1:**
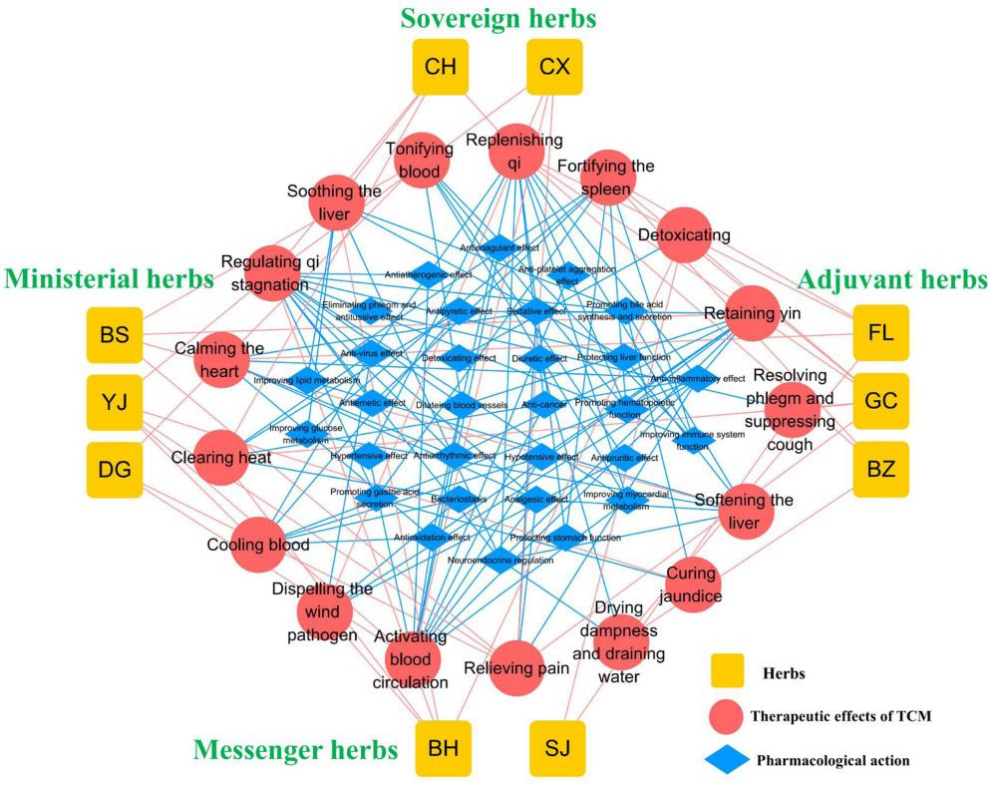
**Overview of TGLQ prescription.** Yellow nodes refer to the ten herbs contained in TGLQ; Red nodes refer to the therapeutic effects of TCM; Blue nodes refer to the pharmacological actions of TGLQ. Red lines refer to the relations between herbs and the therapeutic effects of TCM. Blue lines refer to the relations between the therapeutic effects of TCM and the pharmacological actions of TGLQ.

According to the TCM theory, the ten herbs in TGLQ can be classified as sovereign, ministerial, adjuvant and messenger herbs. CH and CX, as sovereign herbs, play the main roles in the TCM therapeutic effects of soothing the liver, regulating qi and activating blood circulation, thus mainly corresponding to the pharmacological mechanisms of anti-inflammation, anti-platelet aggregation, improving lipid and glucose metabolism, improving the immune system and antiatherogenic effects. BS, DG and YJ, as ministerial herbs, enhance the TCM therapeutic effects of sovereign herbs. In addition, BS and DG can tonify blood, corresponding to the enhancement of immune-system function and the promotion of hematopoietic function. FL, BZ and GC, as adjuvant herbs, play the main roles in the TCM therapeutic effects of fortifying the spleen, replenishing qi and drying dampness, corresponding to the pharmacological mechanisms of protecting stomach function, improving immune-system function, improving lipid and glucose metabolism, regulating the neuroendocrine system and diuretic effects. BH and SJ, as messenger herbs, guide herbs into the associated meridians. The BH TCM therapeutic effect of soothing the liver is consistent with that of CH, while the BH TCM therapeutic effect of clearing heat is associated with the pharmacological mechanism of antipyretic and anti-inflammatory effects. The SJ TCM therapeutic effects of resolving phlegm and suppressing coughs are associated with the pharmacological mechanism of eliminating phlegm and antitussive effects (Figure 1).

To investigate further the associations of each herb’s putative targets with signalling pathways, functional enrichment analysis was performed by TCMIP. The putative targets of herbs contained in TGLQ were enriched in the numbers of AS-related signalling pathways, among which metabolism-related signalling pathways (such as PPARA-activated gene expression, activation of gene expression by SREBF /SREBP, recycling of bile acids and salts, etc.) were the most common. The immune-system and inflammation-related signalling pathway is another form of crucial enriched pathway (such as interleukin signalling, the HSP90 chaperone cycle for steroid hormone receptors, the nuclear-receptor transcription pathway, etc.). Detailed information regarding the above is provided in Supplementary Table 5.

### Atherosclerosis-associated therapeutic targets

**Figure 2:**
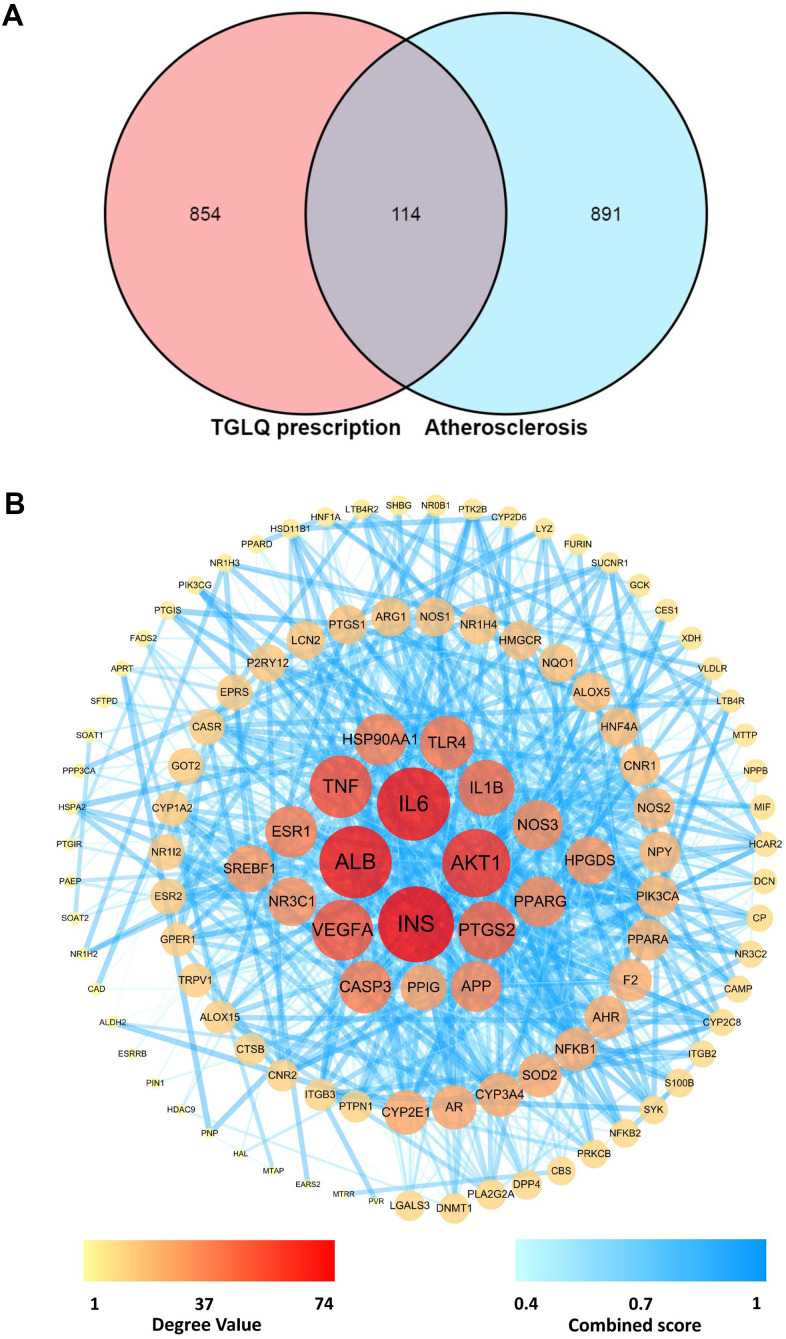
**Acquirement of the key targets between TGLQ putative targets and AS therapeutic targets through network analysis.** (**A**) Venn diagram of common targets of TGLQ and AS; (**B**) Acquirement of the 63 key targets between TGLQ and AS by network topological analysis.

The 1005 therapeutic targets for the treatment of AS were obtained from DisGeNET, TTD and CTD, following the methods cited above. To investigate the underlying mechanisms of TGLQ when acting on AS, the 114 common targets were acquired by intersecting the TGLQ putative targets with AS therapeutic targets, by means of a Venn diagram (Figure 2A).

### Network analysis

The network of 114 common targets was constructed, and then subjected to network topological analysis by the STRING 11.0 database. The network consisted of 114 nodes and 1030 edges, while the average node degree was 18.1. On the basis of network topological analysis and the screening principle mentioned above, the 63 key targets, whose topological-feature values (degree, node betweenness and closeness) were greater than the median, were selected (Figure 2B). Detailed information regarding the 1005 therapeutic targets of AS, and the 114 common targets, is provided in Supplementary Tables 3, 4, respectively.

### Functional enrichment analysis

**Figure 3:**
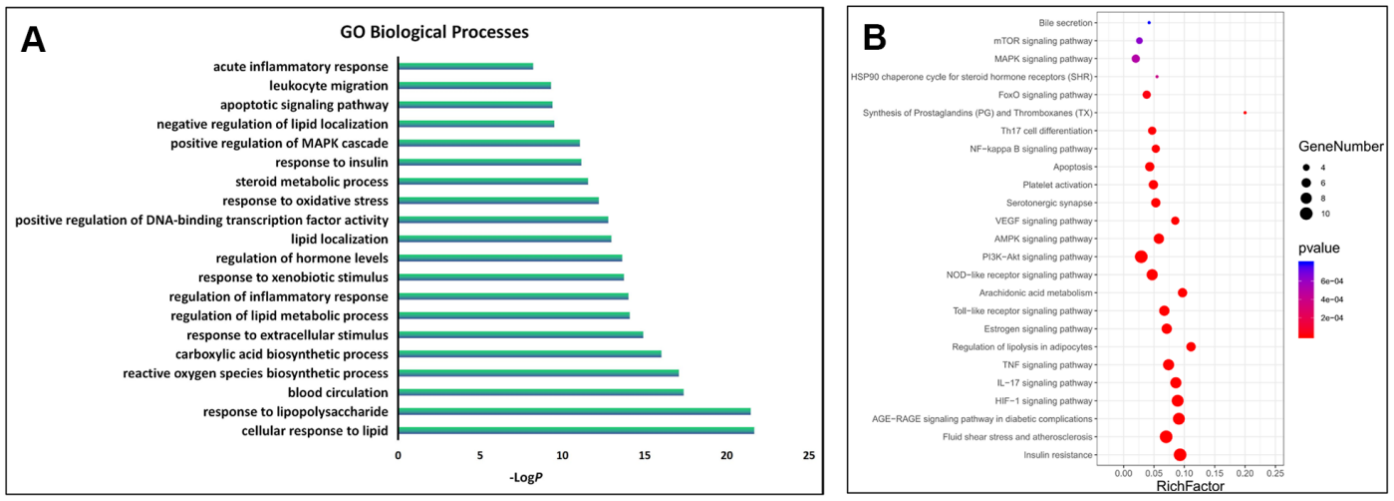
**GO and KEGG enrichment analysis of the key targets.** (**A**) The top 20 significantly enriched terms in the GO biological processes; (**B**) The top 25 significantly enriched terms in KEGG pathways.

**Figure 4:**
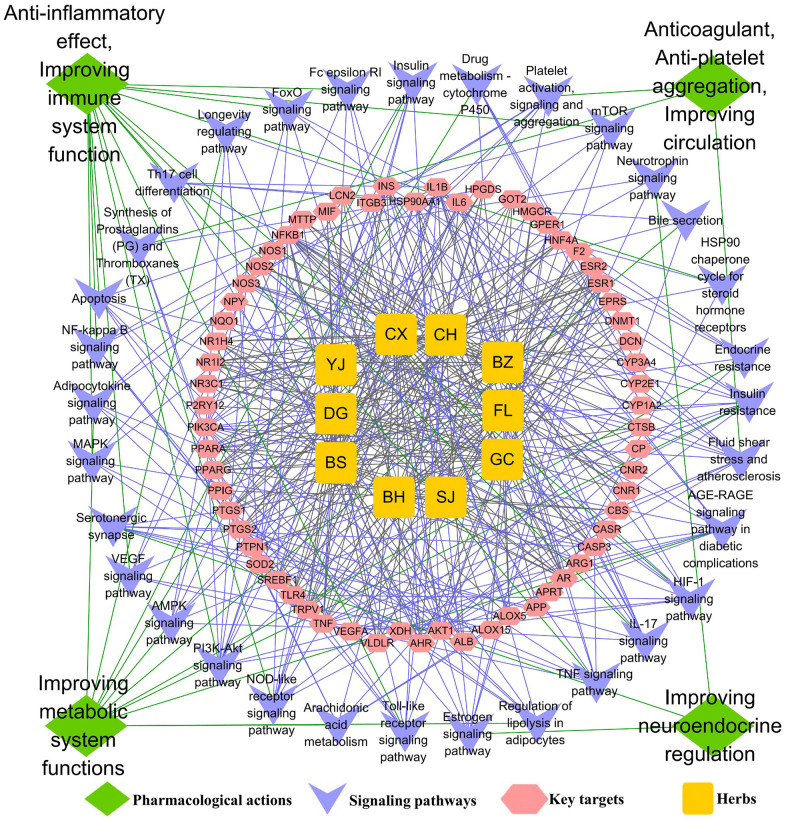
**The underlying mechanisms of TGLQ treatment of AS, via network analysis.** Illustration of the relevance among herbs of TGLQ, their key targets for AS therapy, pathways involved in these key TGLQ targets and the corresponding pharmacological actions. Yellow nodes refer to the ten herbs contained in TGLQ; Red nodes refer to the key targets; Purple nodes refer to the signalling pathways; Green nodes refer to the pharmacological actions. Green lines refer to the relations between the pharmacological actions and the signalling pathways. Purple lines refer to the relations between the signalling pathways and the key targets. Grey lines refer to the relations between the key targets and ten herbs.

The GO biological processes and signalling pathways of the 63 key targets were further subjected to enrichment analysis via the Metascape platform. The top 20 GO biological processes were involved in three aspects of treating AS, including regulating the immune system and inflammation, improving lipid and glucose metabolism, and regulating the neuroendocrine system (Figure 3A). In addition, the top 25 signalling pathways were involved with four main aspects, including regulating the immune system and inflammation, improving lipid and glucose metabolism, regulating the neuroendocrine system and anti-thrombosis effect. Detailed enrichment analysis results for the signalling pathways are shown in Figure 3B, and the network of “herbs-key targets-corresponding signalling pathways-anti-AS pharmacological mechanisms” is shown as Figure 4.

The functional enrichment analysis above indicated that both the GO biological process and the pathway were closely associated with inflammation and lipid metabolism. Additionally, more and more studies have confirmed that, in the progression of AS, the formation of foam cells derived from macrophage was strongly involved with inflammatory response and lipid accumulation. As indicated by the results of the network analysis shown above, TGLQ played a protective role in AS, mainly through the regulation of inflammatory response and lipid metabolism (Figure 4). According to previous studies, TNF-α, IL-1β, IL-6 secreted by ox-LDL induced macrophages are critical pro-inflammatory factors in the development of AS [3, 8]. NR3C1 (nuclear receptor subfamily 3, group C member 1), also called the glucocorticoid receptor (GR), was associated with the lipid accumulation of ox-LDL induced macrophages [14]. Furthermore, these were also the key predicted targets of TGLQ in the treatment of AS. Heat-shock protein 90 (HSP90) and toll-like receptor 4 (TLR4) were the other key predicted targets. In one recent study, the production of HSP90 protein, which could aggravate inflammatory responses and accelerate lipid accumulation in macrophages, was higher in foam cells derived from ox-LDL induced macrophages [14]. TLR4 was a type of pattern-recognition receptor, which could be up-regulated by ox-LDL and could also mediate inflammatory activation [8].

### Effects of TGLQ on AS model ApoE^-/-^ mice

### *TGLQ inhibited AS plaque formation in the aorta of ApoE*^−/−^ *mice*

**Figure 5:**
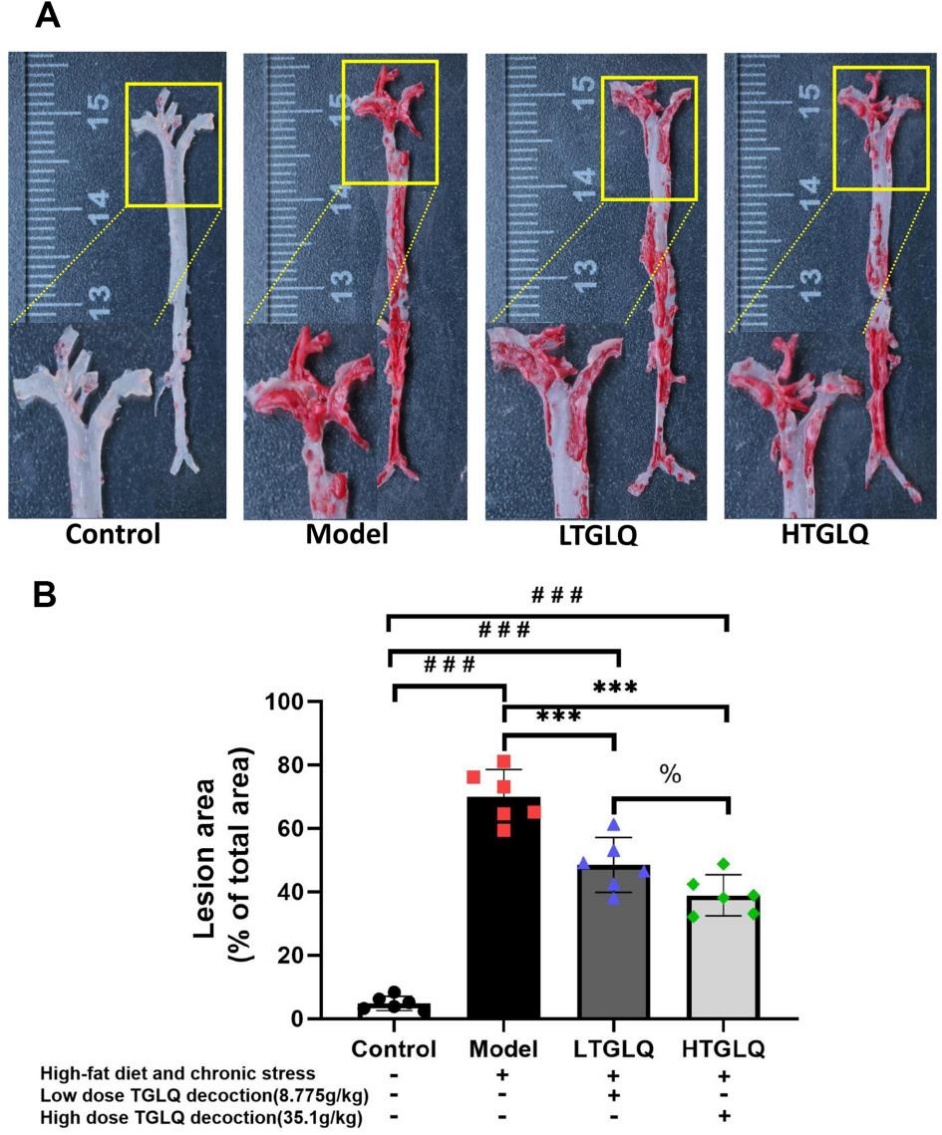
**Oil red O staining of the whole aortic area.** (**A**) Oil red O staining of the whole aortic area; (**B**) represents the percentage of measured lesion area. Oil red O staining to evaluate the severity of the lesions and quantify the atherosclerotic burden; the surface area of the oil red O-positive lesions on the surface of whole aorta was measured. Control group mice were fed with ordinary chow, while mice of the model group, the low-dose TGLQ-prescription (LTGLQ) group and the high-dose TGLQ prescription (HTGLQ) group were intragastrically injected with high-fat emulsion and stimulated with chronic stress. LTGLQ and HTGLQ group mice were intragastrically injected with 8.775g/kg and 35.1g/kg TGLQ prescription daily respectively. #, ##, and ### represent *P* < 0.05, *P* < 0.01, and *P* < 0.001 respectively, in comparison with the control group. *, **, and *** represent *P* < 0.05, *P* < 0.01, and *P* < 0.001 respectively, in comparison with the model group. % represents *P* < 0.05, in comparison with the LTGLQ group.

To investigate the pathogenesis of atherosclerosis, we evaluated AS plaque-lesion formation in the experimental AS model ApoE−/− mice by oil red O staining. As shown in Figure 5, oil red O staining showed that there were significantly increased plaque-lesion areas of aorta in the model group compared with those in the control group (P < 0.001). Moreover, the addition of TGLQ to the AS model ApoE-/- mice (the LTGLQ and HTGLQ group) significantly decreased the plaque-lesion areas of aorta compared with the model group (all P < 0.001). The HTGLQ group evinced a significant decrease in the plaque-lesion areas of aorta compared with the LTGLQ group (P < 0.05). Therefore, the results above suggested that TGLQ could inhibit AS plaque formation in the aorta of experimental AS model ApoE−/− mice.

### *TGLQ decreased the plasma lipid profiles in AS model ApoE*^-/-^ *mice*

**Figure 6:**
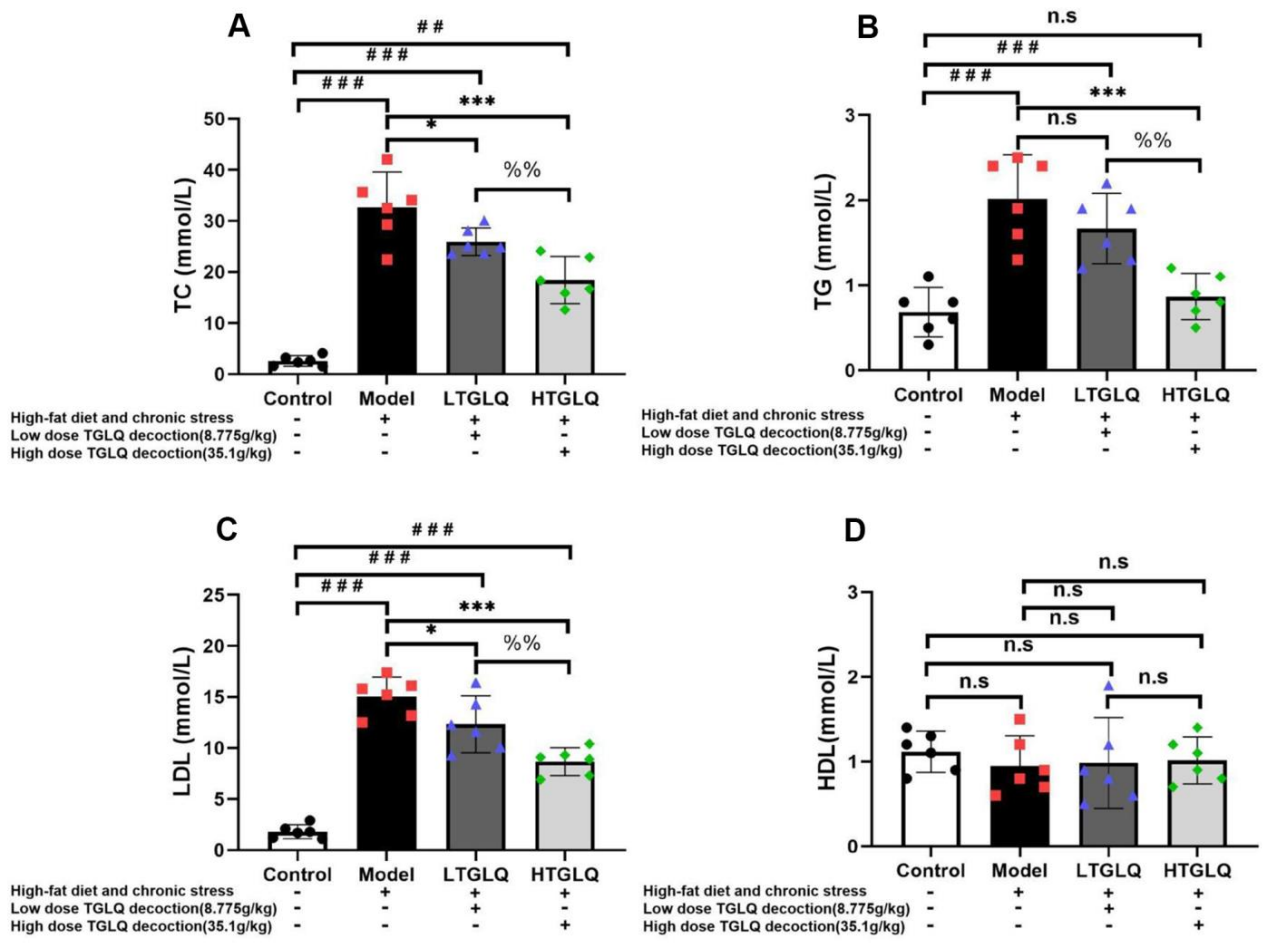
**TGLQ decreased the plasma lipid profiles in AS model ApoE-/- mice.** (**A**–**D**) represent the plasma levels of total cholesterol (TC), triglycerides (TG), low-density lipoprotein (LDL) cholesterol and high-density lipoprotein (HDL) cholesterol in each group, respectively. The plasma levels of TC, TG, LDL and HDL were measured using an autoanalyser. Control-group mice were fed with ordinary chow, while the mice of the model group, the low-dose TGLQ-prescription (LTGLQ) group and the high-dose TGLQ-prescription (HTGLQ) group were intragastrically injected with high-fat emulsion and stimulated with chronic stress. LTGLQ and HTGLQ group mice were intragastrically injected with 8.775g/kg and 35.1g/kg TGLQ prescription daily, respectively. #, ##, and ### represent *P* < 0.05, *P* < 0.01, and *P* < 0.001 respectively, in comparison with the control group. *, **, and *** represent *P* < 0.05, *P* < 0.01, and *P* < 0.001 respectively, in comparison with the model group. %% represents *P* < 0.01, in comparison with the LTGLQ group. n.s represents *P* > 0.05.

In order to explore the lipid metabolism, we detected the plasma levels of TC, TG, LDL and HDL. As shown in Figure 6, the plasma levels of TC, TG and LDL increased significantly in the model group compared with those in the control group (all P < 0.001). Furthermore, the addition of low-dose TGLQ to AS model ApoE-/- mice significantly reduced the levels of TC and LDL, compared with the model group (all P < 0.05). Moreover, the addition of high-dose TGLQ to AS model ApoE-/- mice significantly decreased the levels of TC, TG and LDL compared with the model group (all P < 0.001). Additionally, the HTGLQ group saw significant reductions in the levels of TC, TG and LDL compared with the LTGLQ group (all P < 0.01). There was no significant difference in the levels of HDL among all groups (all P > 0.05). Consequently, the results above suggested that TGLQ could decrease the plasma levels of TC, TG and LDL in AS model ApoE-/- mice (Figure 6).

### *TGLQ suppressed inflammatory cytokines in AS model ApoE*^-/-^ *mice*

**Figure 7:**
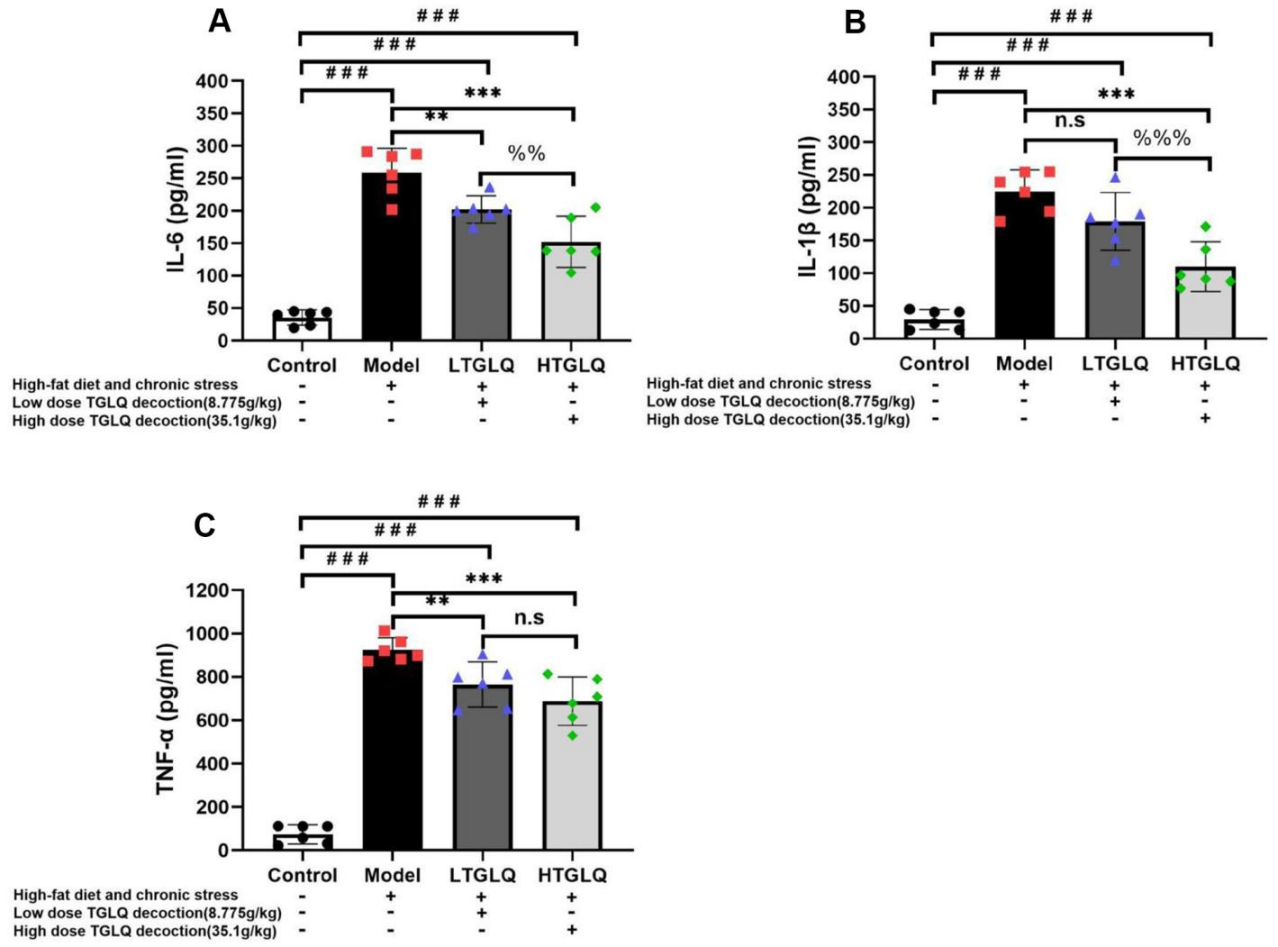
**TGLQ suppressed inflammatory cytokines in AS model ApoE-/- mice.** (**A**–**C**) represent the expression levels of inflammatory cytokines including interleukin (IL)-6, IL-1β, and tumour necrosis factor (TNF)-α detected by ELISA kit respectively, in each group. The control group mice were fed with ordinary chow, while the mice of the model group, the low-dose TGLQ-prescription (LTGLQ) group and the high-dose TGLQ-prescription (HTGLQ) group were intragastrically injected with high-fat emulsion and stimulated with chronic stress. The LTGLQ and HTGLQ group mice were intragastrically injected with 8.775g/kg and 35.1g/kg TGLQ prescription daily respectively. #, ##, and ### represent *P* < 0.05, *P* < 0.01, and *P* < 0.001 respectively, in comparison with the control group. *, **, and *** represent *P* < 0.05, *P* < 0.01, and *P* < 0.001 respectively, in comparison with the model group. %% and %%% represent *P* < 0.01 and *P* < 0.001 respectively, in comparison with the LTGLQ group. n.s represents *P* > 0.05.

In order to investigate the inflammatory response, we detected the plasma levels of TNF-α, IL-1β and IL-6. As shown in Figure 7, the plasma levels of TNF-α, IL-1β and IL-6 increased significantly in the model group compared with those in the control group (all P < 0.001). Furthermore, the addition of low-dose TGLQ to AS model ApoE-/- mice significantly reduced the levels of TNF-α and IL-6 compared with the model group (all P < 0.01). Moreover, the addition of high-dose TGLQ to AS model ApoE-/- mice significantly reduced the levels of TNF-α, IL-1β and IL-6 compared with the model group (all P < 0.001). Additionally, the HTGLQ group evinced a significant reduction in the levels of IL-6 and IL-1β compared with the LTGLQ group (IL-1β, P < 0.01; IL-6, P < 0.001). Thus, the results above suggested that TGLQ could suppress the plasma levels of TNF-α, IL-1β and IL-6 in AS model ApoE-/- mice.

### Effects of TGLQ on ox-LDL induced macrophages

### TGLQ increased the viability of ox-LDL induced macrophages

**Figure 8:**
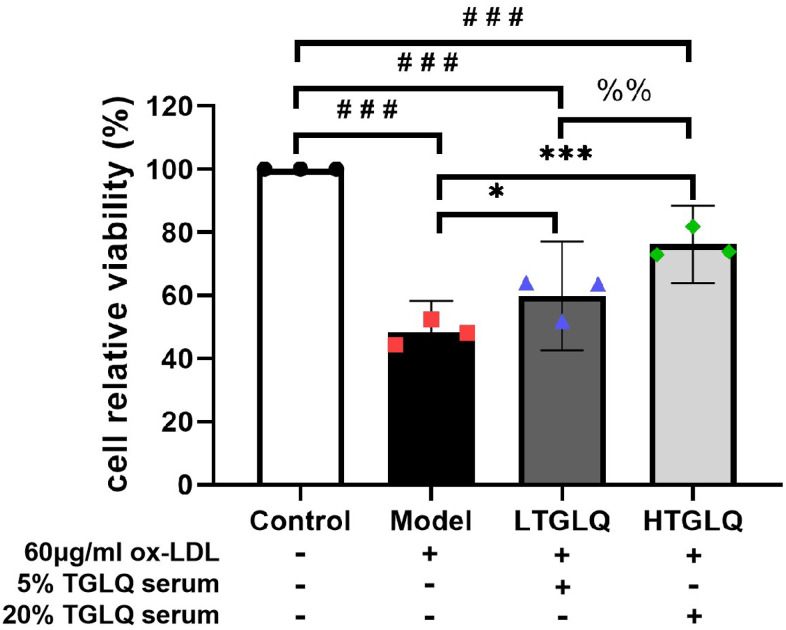
**TGLQ increased the viability of ox-LDL induced macrophages.** The viability of macrophages was determined by using a CCK-8 kit. Unlike the macrophages of the control group, the macrophages of the model group, the low-dose TGLQ-serum (LTGLQ) group and the high-dose TGLQ-serum (HTGLQ) group were exposed to 60μg/ml oxidized-LDL (ox-LDL) for 24 h. In addition, control serum, 5% TGLQ serum and 20% TGLQ serum were added to the model group, the LTGLQ group and the HTGLQ group, respectively. #, ##, and ### represent *P* < 0.05, *P* < 0.01, and *P* < 0.001 respectively, in comparison with the control group. *, **, and *** represent *P* < 0.05, *P* < 0.01, and *P* < 0.001 respectively, in comparison with the model group. %% represents *P* < 0.01, in comparison with the LTGLQ group.

We used CCK-8 assay to explore the effects on the relative viability of macrophages. According to our CCK-8 assay results, the relative viability of macrophages in the model group significantly decreased compared with that in the control group (P < 0.001). As presented in Figure 8, the LTGLQ group (P < 0.05) and the HTGLQ group (P < 0.001) showed significantly higher viability of macrophages when compared with the model group. Moreover, the relative viability of macrophages in the HTGLQ group significantly increased compared with that in the LTGLQ group (P < 0.01). These results implied that TGLQ could relieve cellular injury induced by ox-LDL in macrophages.

### TGLQ reduced the accumulation of lipid in ox-LDL induced macrophages

**Figure 9:**
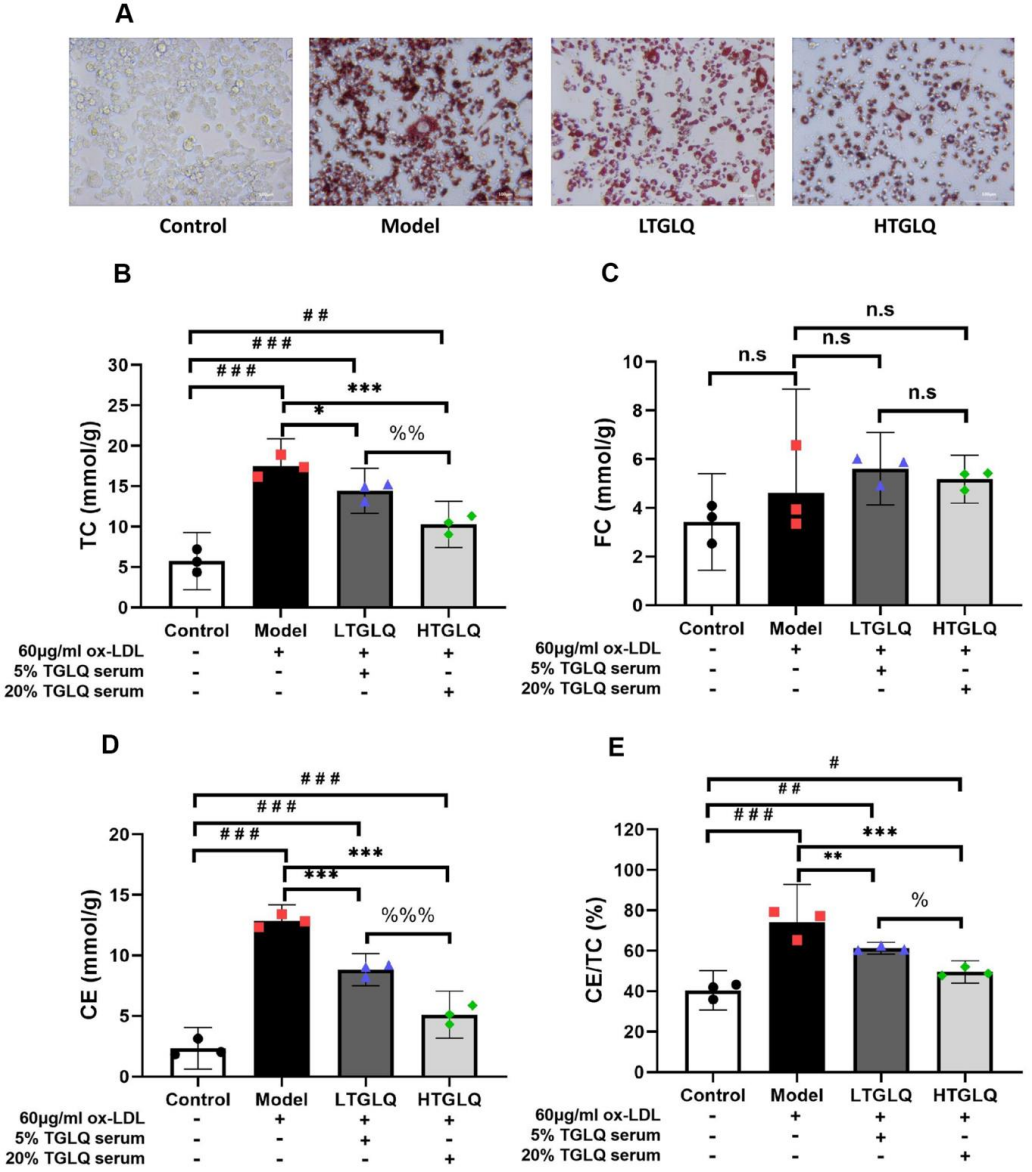
**TGLQ reduced the accumulation of lipid in ox-LDL induced macrophages.** (**A**) Oil red O Staining was applied to estimate the level of lipid accumulation; (**B**–**D**) represent the expression levels of total cholesterol (TC), free cholesterol (FC) and cholesterol ester (CE) in each group respectively. The levels of TC, FC and CE in the culture medium were detected by using commercial kits. (**E**) Percentage of TC to CE ratio in each group. Unlike the macrophages of the control group, the macrophages of the model group, the low-dose TGLQ-serum (LTGLQ) group and the high-dose TGLQ-serum (HTGLQ) group were exposed to 60μg/ml oxidized-LDL (ox-LDL) for 24 h. In addition, control serum, 5% TGLQ serum and 20% TGLQ serum were added to the model group, the LTGLQ group and the HTGLQ group, respectively. #, ##, and ### represent *P* < 0.05, *P* < 0.01, and *P* < 0.001 respectively, in comparison with the control group. *, **, and *** represent *P* < 0.05, *P* < 0.01, and *P* < 0.001 respectively, in comparison with the model group. %, %% and %%% represent *P* < 0.05, *P* < 0.01 and *P* < 0.001 respectively, in comparison with the LTGLQ group. n.s represents *P* > 0.05.

To investigate the accumulation of lipid, we conducted oil red O staining, and detected the levels of TC, FC and CE in a culture medium. Oil red O staining showed that there was a large amount of red staining lipid accumulation in the model group, compared with the control group (Figure 9A). In addition, TC, CE and CE/TC (> 50%) were increased significantly in the model group compared with their respective levels in the control group (all P < 0.001). Furthermore, the results of oil red O staining showed that the addition of TGLQ to macrophages treated with ox-LDL (the LTGLQ and HTGLQ group) significantly decreased lipid accumulation compared with the model group. Moreover, compared with the model group, the levels of TC, CE and CE/TC were decreased significantly in the LTGLQ group (TC, P < 0.05; CE, P < 0.001; CE/TC, P < 0.01) and the HTGLQ group (all P < 0.001). Additionally, compared with the LTGLQ group, the levels of TC, CE and CE/TC were decreased significantly in the HTGLQ group (TC, P < 0.01; CE, P < 0.001; CE/TC, P < 0.05). Therefore, the results above suggested that TGLQ could reduce intracellular lipid accumulation of macrophages induced by ox-LDL (Figure 9B–9E).

### TGLQ suppressed inflammatory cytokines in ox-LDL induced macrophages

**Figure 10:**
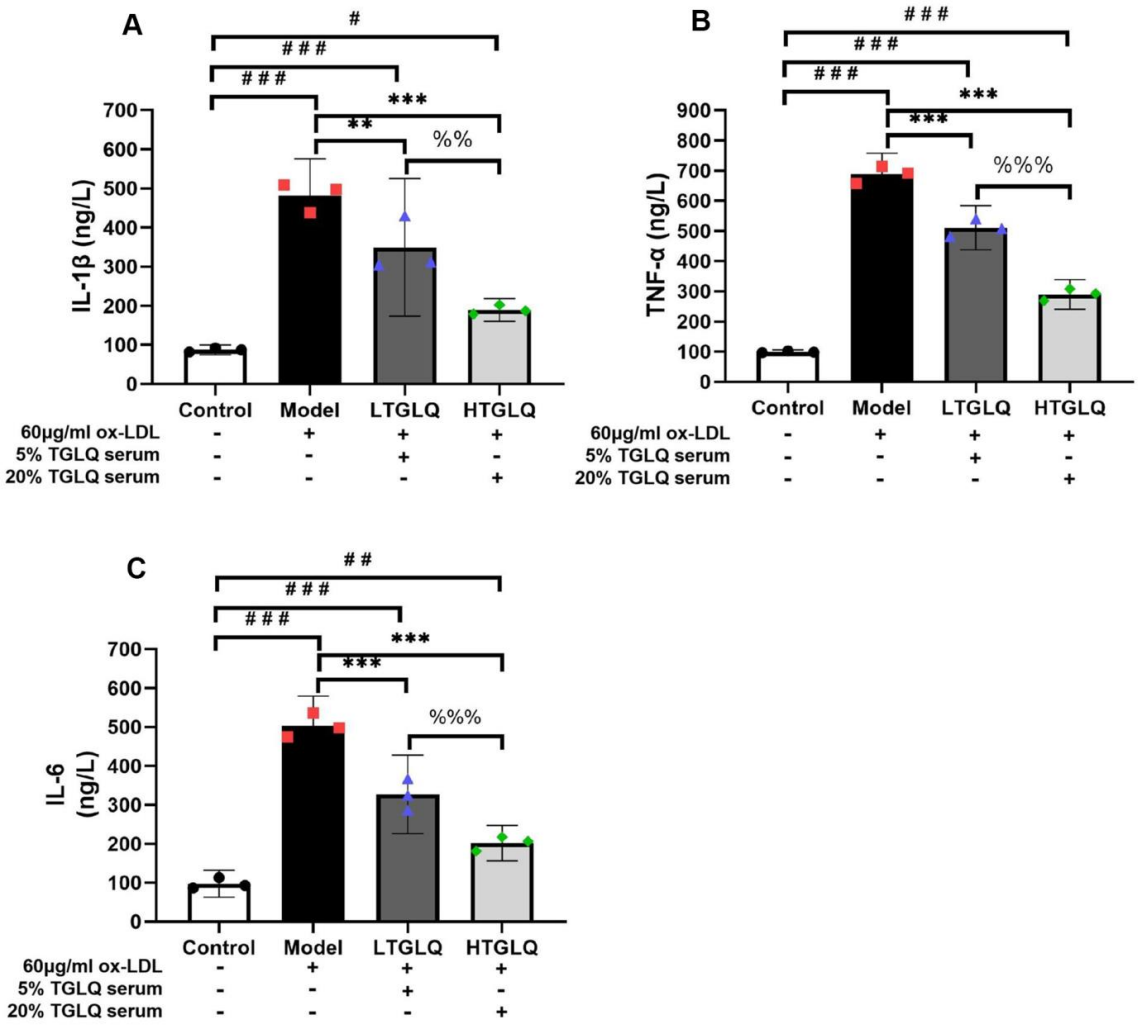
**TGLQ suppressed inflammatory cytokines in ox-LDL induced macrophages.** (**A**–**C**) represent the expression levels of inflammatory cytokines, including interleukin-1β (IL-1β), tumour necrosis factor-α (TNF-α) and interleukin-6 (IL-6) respectively in each group. The expression levels of IL-1β, TNF-α and IL-6 in the culture medium were detected by ELISA kits. Unlike the macrophages of the control group, the macrophages of the model group, the low-dose TGLQ-serum (LTGLQ) group and the high-dose TGLQ-serum (HTGLQ) group were exposed to 60μg/ml oxidized-LDL (ox-LDL) for 24 h. In addition, control serum, 5% TGLQ serum and 20% TGLQ serum were added to the model group, the LTGLQ group and the HTGLQ group, respectively. #, ##, and ### represent *P* < 0.05, *P* < 0.01, and *P* < 0.001 respectively, in comparison with the control group. *, **, and *** represent *P* < 0.05, *P* < 0.01, and *P* < 0.001 respectively, in comparison with the model group. %% and %%% represent *P* < 0.01 and *P* < 0.001 respectively, in comparison with the LTGLQ group.

In order to investigate the inflammatory response, we detected the levels of TNF-α, IL-1β and IL-6 in a culture medium. The results showed that, compared with the control group, the levels of IL-1β, TNF-α, and IL-6 were significantly increased in the model group (all P < 0.001). Moreover, results demonstrated that the addition of low-dose TGLQ treatment to macrophages treated with ox-LDL significantly decreased the levels of IL-1β, TNF-α and IL-6, as compared with the model group (LTGLQ group: IL-1β, P < 0.01; TNF-α, P < 0.001; IL-6, P < 0.001). Meanwhile, the addition of high-dose TGLQ treatment to macrophages treated with ox-LDL significantly decreased the levels of IL-1β, TNF-α and IL-6 compared with the model group (all P < 0.001). Furthermore, compared with the LTGLQ group, the levels of IL-1β, TNF-α and IL-6 were decreased significantly in the HTGLQ group (IL-1β, P < 0.01; TNF-α, P < 0.001; IL-6, P < 0.001). The results above indicated that TGLQ could suppress the production of TNF-α, IL-1β and IL-6 in macrophages stimulated by ox-LDL (Figure 10A–10C).

### TGLQ inhibited protein expression of HSP90 and TLR4 in ox-LDL induced macrophages

**Figure 11:**
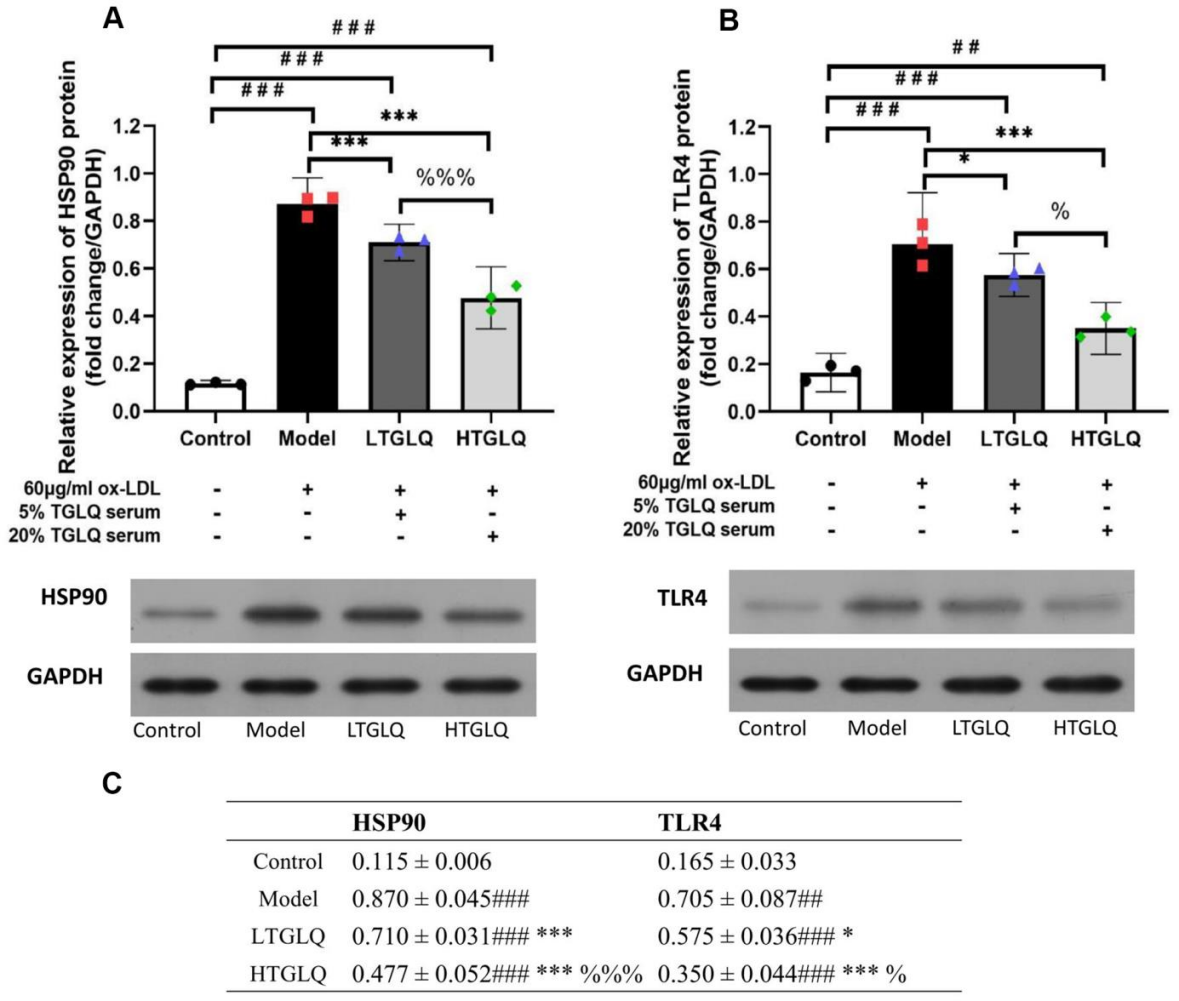
**TGLQ inhibited protein expression of HSP90 and TLR4 in ox-LDL induced macrophages.** (**A**, **B**) represent the protein expression levels of HSP90 and TLR4 detected by western blot, respectively, in each group. (**C**) represents data shown in Figure 11A, 11B. Unlike the macrophages of the control group, the macrophages of the model group, the low-dose TGLQ-serum (LTGLQ) group and the high-dose TGLQ-serum (HTGLQ) group were exposed to 60μg/ml oxidized-LDL (ox-LDL) for 24 h. In addition, control serum, 5% TGLQ serum and 20% TGLQ serum were added to the model group, the LTGLQ group and the HTGLQ group, respectively. #, ##, and ### represent *P* < 0.05, *P* < 0.01, and *P* < 0.001 respectively, in comparison with the control group. *, **, and *** represent *P* < 0.05, *P* < 0.01, and *P* < 0.001 respectively, in comparison with the model group. % and %%% represent *P* < 0.05 and *P* < 0.001 respectively, in comparison with the LTGLQ group.

On the basis of the network analysis results described above, we validated the potential mechanisms by detecting the HSP90 and TLR4 protein expression. In this study, compared with the control group, the protein expressions of HSP90 and TLR4 were significantly increased in the model group (all P < 0.001). Moreover, compared with the model group, the protein expressions of TLR4 and HSP90 were significantly decreased in the LTGLQ group (HSP90, P < 0.001; TLR4, P < 0.05) and the HTGLQ group (all P < 0.001). Furthermore, compared with the LTGLQ group, the protein expressions of TLR4 and HSP90 were also significantly decreased in the HTGLQ group (HSP90, P < 0.001; TLR4, P < 0.05). These findings suggested that protective effects of TGLQ on ox-LDL induced macrophages were closely related to the inhibition of TLR4 and HSP90 expression (Figure 11A, 11B).

## DISCUSSION

Within this study, network pharmacological analysis provided a potential means to investigate systematically the underlying mechanisms of TGLQ in its action on AS. Furthermore, we conducted *in vivo* and *in vitro* experiments to validate the effects of TGLQ on AS models and several predicted targets.

The development of AS is strongly associated with abnormal lipid metabolism and inflammatory response, which are the main pathological mechanisms, and thus the current focus of medical development. According to TCM theory, the aetiology and pathogenesis of AS are summarized as “qi stagnation and blood stagnation”, which is related to “liver dysfunction”. Thus, the TCM treatment principle of “improving the liver function”, “regulating qi stagnation” and “activating blood circulation” (Tiaogan-Liqi method) have been applied widely in clinical settings. Modified Xiaoyaosan (Tiaogan-Liqi prescription, TGLQ), as representative of the TCM treatment principle above, has provided an alternative therapy for the management of AS in Asian countries [18]. The results of our previous study demonstrated that Xiaoyaosan, which was the prototype for TGLQ, could inhibit the development of AS in high-fat fed ApoE-/- mice by down-regulating the levels of blood lipid and inflammatory cytokines [14]. In addition, certain studies showed similar protective effects for TCM formula, which were based on the same TCM treatment principle, acting upon AS [8, 19]. Ligusticum chuanxiong Hort. (Chuanxiong, CX) and Curcuma wenyujin Y. H. Chen et C. Ling (Yujin, YJ) played important roles in enhancing the effects of “regulating qi stagnation” and “activating blood circulation” in TGLQ. A growing number of studies have confirmed the anti-inflammatory, anti-thrombotic and anti-atherosclerosis effects of CX [9, 20]. Furthermore, a combination of CX and YJ could significantly suppress the development of AS in Western-diet fed ApoE-/- mice by regulating oxidative stress, metabolism and inflammatory response [11, 21] (Figure 4).

The positive results of the studies above confirmed only a part of the effects of CHM formulas, however. It was difficult to expound completely the mechanism of CHM formulas in treating AS by a conventional pharmacological approach. Network pharmacology, as a part of systems-biology methodology, provides an efficient and comprehensive approach for revealing the holistic pharmacological effects of TCM prescriptions at the molecular level. The methodology shifts the TCM pharmacological research model from “one drug, one target” to “multi-component therapeutics, biological networks”, which accords with the holism and complexity of TCM characteristics. Therefore, we first applied TCMIP, a network pharmacological platform, to collect and analyse the putative targets of the ten herbs in TGLQ. The results showed that there were a large number of common putative targets of the ten herbs contained in TGLQ (Table 1), indicating potential herb-herb interactions through these common targets. Combining the putative targets of TGLQ with the therapeutic targets of AS, we screened 63 key targets according to the results of network topological analysis. Moreover, functional enrichment analysis of these 63 key targets showed that TGLQ might inhibit AS by regulating the immune system and inflammation, improving lipid and glucose metabolism, and regulating the neuroendocrine system and anti-thrombosis effects (Figure 4). Among the results above, we have validated the hypothesis that the prototype formula of TGLQ could inhibit the formation of atherosclerotic plaque of high-fat fed ApoE-/- mice by regulating NR3C1 (nuclear receptor subfamily 3, group C member 1), also called the glucocorticoid receptor (GR), which was associated with improving the lipid metabolism [14, 22]. In this study, it was striking that not only could TGLQ improve lipid metabolism, but it could also suppress the inflammatory response (Figures 7, 10). In addition, the inflammatory cytokines (TNF-α, IL-1β and IL-6) and related targets (HSP90 and TLR4) were at the top of the list (so to speak) of key targets, which indicated that TGLQ might be used to treat AS by regulating these targets (Figure 2B).

In this study, and on the basis of the network analysis results above, we carried out *in vivo* and *in vitro* experiments to validate the effects of TGLQ on AS model ApoE^-/-^ mice, and on macrophages induced by ox-LDL, respectively. The results of *in vivo* experiments validated the hypothesis that TGLQ could significantly decrease plasma lipid profiles and plasma inflammatory cytokines (TNF-α, IL-1β and IL-6), and inhibit the AS plaque formation of the AS model ApoE^-/-^ mice. Ox-LDL, as a damage-associated molecular pattern molecule, is phagocytosed by macrophages, which subsequently leads to the production of inflammatory cytokines. Accordingly, we conducted *in vitro* experiments, and the results showed that TGLQ could significantly reduce intracellular lipid accumulation, and also suppress the production of inflammatory cytokines of macrophages induced by ox-LDL. Furthermore, the expression of TLR4 and HSP90 in ox-LDL induced macrophages was significantly inhibited by TGLQ, implying that TGLQ had the potential to regulate signalling pathways related to TLR4 and HSP90, although this possibility requires further study and development.

Data from numerous studies have confirmed the significant role of inflammatory cytokines, such as TNF-α, IL-6 and IL-1β, in the development of AS. Many studies have provided strong evidence that IL-1β is a pro-atherogenic factor, and increased levels of IL-1β were observed in atherosclerotic lesions, associated with the severity of the disease [23, 24]. Besides this, it has been proven that high levels of IL-1β are associated with an increased incidence of cardiovascular events in AS patients who evince atherosclerotic risk factors [25, 26]. The Canakinumab Anti-Inflammatory Thrombosis Outcomes Study (CANTOS trial) proved that inhibition of IL-1β had a beneficial effect on the progression of AS [27]. Both TNF-α and IL-6 have been widely investigated, not only from a pathophysiological perspective, but also as therapeutic targets for AS [28, 29]. TNF-α has been known to stimulate the synthesis of IL-6 during the process of immune activation and inflammation, which aggravates the pathological process of AS [30]. The cellular effects of TNF-α were primarily mediated by the p38 mitogen-activated protein kinase (p38MAPK)/ nuclear factor kappa-lightchain-enhancer of the activated B-cell (NF-κB) pathways. The effects of IL-6 were mediated by the IL-6 receptor and the signal transducer protein gp130 [31].

TNF-α significantly impaired vascular health by mediating inflammation, which increased reactive oxygen species and promoted the adhesion of leukocytes to the vascular wall, inducing cell apoptosis [32]. In addition, previous epidemiological studies demonstrated that higher serum TNF-α concentrations were related to higher risk of ischemic stroke and recurrent coronary events [33, 34]. Heat-shock protein 90 (HSP90) is a ubiquitous molecular chaperone that is involved in signal transduction and transcriptional regulation. Moreover, HSP90 could fold and stabilize many client proteins involved in cell proliferation, apoptosis and inflammation; therefore, it has been proposed as an important target in terms of immunity and inflammation [35]. HSP90 was abundantly expressed in the cytoplasm of eukaryotic cells, and was recently reported to promote AS *in vitro* and *in vivo* [36]. A recent study demonstrated that overexpression of HSP90 resulted in a decrease in AS plaque stability, along with increased foam cells and inflammatory cytokines [36]. Moreover, emerging research shows that the production of HSP90 protein is highly expressed in foam cells derived from ox-LDL induced macrophages, further validating the positive involvement of HSP90 in the development of AS [37].

Shimp et al. reported that suppressing HSP90 could decrease the expression levels of pro-inflammatory mediators by inhibiting the activation of NF-κB pathways [38]. In addition, functional enrichment analysis in the present study showed that HSP90 was involved in several inflammatory response-related signalling pathways, including the NOD-like receptor, PI3K-Akt, IL-17, Th17 cell-differentiation and NF-κB signalling pathways. TLR4, a type of pattern-recognition receptor (PRR) mediating inflammatory activation, can identify endogenous ligands such as ox-LDL and HSPs. TLR4 was often released in response to stress or tissue damage at sites of chronic inflammation, such as AS [39]. It has been reported that TLR4, which is essential for ox-LDL induced macrophage inflammation and differentiation into foam cells, was increased in macrophages of lipid-rich atherosclerotic lesions. This was probably due to ox-LDL acting as a ligand for TLR4, and eventually activating the NF-κB transcription factor, which leads to the synthesis of inflammatory cytokines and chemokines [40, 41]. More and more studies have demonstrated that inhibition of TLR4 signal transduction is beneficial in preventing the development of AS by decreasing levels of inflammatory cytokines (TNF-α, IL-1β and IL-6) [42, 43]. Furthermore, functional enrichment analysis in the present study indicated that TLR4 was involved in certain inflammatory response-related signalling pathways, including the toll-like receptor, HIF-1, NOD-like receptor, PI3K-Akt and NF-κB signalling pathways.

In conclusion, the main findings in this study were as follows: (1) A total of 548 chemical compounds contained in TGLQ and 969 putative targets were screened from TCMIP; (2) A total of 1005 therapeutic targets for the treatment of AS were obtained from DisGeNET, TTD and CTD; (3) The 63 key targets were screened by network topological analysis, and functional enrichment analysis showed that the key targets were associated with immune system and inflammation, lipid-and-glucose metabolism, neuroendocrine system and anti-thrombosis effect; (4) The *in vivo* experiments confirmed that TGLQ could significantly decrease plasma lipid profiles and plasma inflammatory cytokines, and inhibit the AS plaque formation of the AS model ApoE^-/-^ mice; (5) The *in vitro* experiments showed that TGLQ could significantly reduce intracellular lipid accumulation, and also suppress the production of the inflammatory cytokines of macrophages, induced by ox-LDL, that were associated with TLR4 and HSP90.

Nonetheless, there were also certain inevitable limitations in this study, as follows: (1) There might be insufficient putative targets in terms of TGLQ, because some components contained in TGLQ have not been investigated; (2) The direct and indirect correlations between the herbs contained in TGLQ and the key targets were not clear; (3) The inhibition and promotion effects of TGLQ on the key targets were indeterminate, needing further validation. Last but not least, moreover, further experimental studies will be needed to validate the findings of this preliminary study.

## MATERIALS AND METHODS

### Prediction of TGLQ-prescription putative targets

The chemical information for the compositive compounds in CH, CX, YJ, DG, BS, BZ, FL, GC, BH and SJ was obtained from the Integrative Pharmacology-based Research Platform of Traditional Chinese Medicine (TCMIP, Version 2.0, http://www.tcmip.cn), which is a computational platform containing the Chemistry Database of the TCM and Drug Target Prediction Tool [15]. TCMIP provides predicted target genes of herbs and formulas according to a high Tanimoto score (> 0.80), which is a criterion based on the structural and chemical similarities of ingredients with known drugs (from Drug Bank). The Tanimoto score ranges from 0–1, where ‘0’ denotes completely different structures between ingredients and known drugs, and where ‘1’ means identical structures of two components. The component-target pairs for which the Tanimoto scores were higher than 0.80, were selected. The selected targets were identified as putative targets of the herbs above (TGLQ prescription).

### Atherosclerosis-associated therapeutic targets

Known therapeutic targets for the treatment of AS were collected from three databases, as follows. The genes whose evidence index of gene-disease association equalled 1 in the DisGeNET database (https://www.disgenet.org/) were collected; the genes associated with “atherosclerosis” in the TTD database (theoretical target database, http://db.idrblab.net/ttd/) and the CTD database (Comparative toxicogenomics Database, http://ctdbase.org/) were collected. Then, the collected genes from the three databases were merged, and the duplication was subsequently deleted.

### Network analysis

To investigate the effects of TGLQ prescription on AS, the putative targets of TGLQ prescription and the therapeutic targets of AS were intersected to obtain the common targets; this was facilitated by the use of a Venn diagram. To evaluate the importance of the common targets, the latter, duly obtained, were imported to the STRING11.0 database (https://string-db.org/) for network topological analysis (combined score > 0.4 was the screening standard). The three network topological features (including “Degree”, “Node betweenness” and “Closeness”) of each common target were calculated through network analysis. The definitions of the three topological features are described as follows: “Degree” refers to the number of interactions to node i; “Node betweenness” refers to the number of the shortest paths between node pairs which run through node i; “Closeness” refers to the sum of the node i distances to all other nodes, and measures how long it will take sequentially to spread information from node i to all the other nodes [44]. The larger the degree/node betweenness/closeness centrality of a given node, the more important it was that that node be included in the network. According to this paradigm, the common targets, whose topological features mentioned above were greater than the median, were screened as the key targets [45]. Cytoscape software (Version 3.6.0) was applied to visualize the interaction network.

### Functional enrichment analysis

Metascape (http://metascape.org/) is a database and platform with appropriate timeliness and accuracy, which integrates Gene Ontology (GO), the Kyoto Encyclopaedia of Genes and Genomes (KEGG), UniProt and other databases. It can be used for GO analysis, KEGG and Reactome signalling-pathway analysis. By uploading the targets to Metascape and setting the parameter as “H species”, the results of the enrichment analysis for the GO biological process and the KEGG signalling-pathway analysis were obtained. Top ranked biological processes and pathways were analysed and screened according to their physiological and pharmaceutical importance. (Omicshare https://www.omicshare.com/tools/) was applied to visualize the results above.

### Experimental validation *in vivo*

We validated the effects of TGLQ on AS and the results of the network analysis above by experiment *in vivo*.

### Materials, animals and *in vivo* experimental design

Animal studies were conducted in compliance with the ARRIVE guidelines, with the approval of the Local Ethical Committee (Guangzhou University of Chinese Medicine). Male C57BL/6 (n = 6, 4 weeks old, weight 17-20g) and male Apolipoprotein E-deficient (ApoE^-/-^) mice (n = 18, 4 weeks old, weight 17-20g, C57BL/6 background) were purchased from the Model Animal Research Centre, Nanjing University, Nanjing, China. Before the beginning of the experiments, all the mice were kept in rooms under controlled laboratory conditions (23-25° C, 12:12h light-dark cycle, 50%-70% humidity) and fed with approved ordinary chow for at least 7 days along with free access to water. In line with our previous AS model construction [14], 30 ApoE^-/-^ mice were intragastrically (ig) injected with 0.2 ml of high-fat emulsion daily (including 10% lard oil, 10% tween-80, 10% propylene glycol, 5% cholesterol, 1% sodium cholate, 1% propylthiouracil, and purified water), and stimulated with chronic stressors (including ice-water swimming for 30s-60s, day and night inversion for 12 h respectively, fettering for 6 h, cage inclination to 45 degrees, water deprivation for 24 h, and food deprivation for 24 h) with a frequency of a six-day cycle, for 16 weeks. On the last day of the 8th week of model construction, the 18 ApoE^-/-^mice were randomly divided into 3 groups (n=6 in each group), including the model group (model, intragastrically injected with 1ml normal saline daily), the low-dose TGLQ group (LTGLQ, intragastrically injected with 8.775g/kg daily) and the high-dose TGLQ group (HTGLQ, intragastrically injected with 35.1g/kg daily). The duration of the TGLQ treatment was 8 weeks.

The TGLQ prescription consisted of 10 Chinese herbal medicines, and the preparation process was as follows. *Bupleurum Chinense DC.* (9 g), *Angelica sinensis (Oliv.) Diels*(9 g), *Paeonia lactiflora Pall.*(9 g), *Atractylodes macrocephala Koidz.*(9 g), *Poriacocos (Schw.) Wolf* (9 g), *Glycyrrhiza uralensis Fisch* (4.5 g), *Mentha haplocalyx Briq.*(4.5 g), *Zingiber officinale Rosc.*(4.5 g), *Ligusticum chuanxiong Hort.*(4.5 g)*, Curcuma wenyujin Y. H. Chen et* (4.5 g) were weighed, soaked for 2 h and extracted in boiling water (1.0 L) twice, for 1 h. The extracted decoction above was combined and dried to powder by decompression. The extract was stored at -80° C and re-dissolved with purified water, before being administered to the mice. The C57BL/6 mice were divided into the control group, which was fed with regular chow and injections of saline. All the mice were sacrificed on the last day of the experimental design, after which the plasma was collected and stored at -80° C. The whole aortas were extracted and embedded in 4% paraformaldehyde for histological analysis.

### Oil red O staining to evaluate the severity of AS plaque

To evaluate the severity of the lesions and to quantify the atherosclerotic burden, the surface area of the Oil Red O-positive lesions on the surface of the whole aorta was measured. The aorta was opened lengthwise and the edges and corners were flattened on black boards. The en face analysis of the lesion area on the whole aorta was quantified based on Oil Red O staining, using Image-Pro Plus 6.0 software (Media Cybernetics, Bethesda, USA) [46].

### Plasma lipid profile analysis

The plasma levels of total cholesterol (TC), triglycerides (TG), high-density lipoprotein (HDL) cholesterol and low-density lipoprotein (LDL) cholesterol were measured using an autoanalyser (Hitachi, Tokyo, Japan).

### Enzyme-linked immunosorbent assay (ELISA)

Plasma samples of mice were prepared for enzyme-linked immunosorbent assay (ELISA). The levels of plasma inflammatory cytokines, including interleukin (IL)-6, IL-1β, and tumour necrosis factor (TNF)-α, were detected using a commercial ELISA kit according to the manufacturer’s instructions. ELISA kits for monitoring IL-6, IL-1β and TNF-α were obtained from Elabscience Biotechnology Co. Ltd. (Wuhan, China). A Thermo microplate reader (MultiskanMK3; Thermo Fisher Scientific Inc., Rockford, IL, USA) was used for the measurement of optical density (OD) at 450 nm.

### Experimental validation *in vitro*

We validated the results of the network analysis by experiment *in vitro*.

### Preparation of TGLQ serum

20 Sprague Dawley (SD) rats (male, age 8 weeks) were randomly divided into control and TGLQ groups. The rats were intragastrically administered saline (n=10) or TGLQ prescription (24.3g/kg, n=10) continuously for 5 days. One hour after the last administration, the rats were anesthetized by intraperitoneal injection with chloral hydrate. Blood was taken from the abdominal aorta and then centrifugated at 4° C, 3000 R / min for 10 min to obtain the control and TGLQ serum. The serum was filtered with a 0.22 μm filter membrane and stored at -80° C for the subsequent experiments.

### Cell culture

RAW264.7 mouse macrophages were obtained from the cell bank of the Chinese Academy of Sciences. The cells were cultured in DMEM containing 10% FBS. The cultured condition was a humidified incubator with 5% CO_2_ and 95% O_2_ at a temperature of 37° C. The cells for subsequent experiments were derived from the second-third passages, when they reached a confluence of 80%.

### Treatment protocol

After 8 h of starvation in serum-free DMEM, the cells were randomly divided into four different groups: a control group, model group, LTGLQ group (low-dose TGLQ treatment group, containing 5% TGLQ serum) and HTGLQ group (high-dose TGLQ treatment group, containing 20% TGLQ serum). Unlike the control group, the other three groups were exposed to 60μg/ml oxidized-LDL (ox-LDL) (Yiyuan Biotechnology, Guangzhou, China) for 24 h as previously indicated [47]. Control serum was added to the model group, while 5% and 20% concentration TGLQ serum were separately added to the LTGLQ group and the HTGLQ group, respectively.

### Cell proliferation assay

The cell proliferation was determined by using a CCK-8 kit (Beyotime, Shanghai, China). RAW264.7 cells were plated in 96-well plates and treated with the corresponding drugs as described above. Subsequently, 10 μl CCK-8 solution was added to each well and incubated for 1 h in an incubator. Then, absorbance at 450 nm was detected by a microplate reader (MultiskanMK3; Thermo Fisher Scientific Inc., Rockford, IL, USA).

### Oil red O staining

Treated cells were plated in a chamber and cultured in an incubator. After 24 h, the medium of treated cells was removed, and the cells were gently rinsed with PBS and fixed with formalin for 15 min. Then, the cells were rinsed with 60% isopropanol and stained with oil red O fluid for 15 min. Subsequently, the cells were washed with distilled water. Finally, the red intracellular lipid droplets were observed through the microscope.

### Test for the content of total cholesterol (TC), free cholesterol (FC) and cholesterol ester (CE)

The levels of total cholesterol (TC), free cholesterol (FC) and cholesterol ester (CE) in the culture medium were detected using commercial kits according to the manufacturer’s instructions (Nanjing Jiancheng Bioengineering Institute, Nanjing, China). A Thermo microplate reader was used for the measurement of optical density (OD) at 450 nm.

### ELISA for inflammatory cytokines

The levels of inflammatory cytokines, including tumour necrosis factor-α (TNF-α), interleukin-1β (IL-1β), and interleukin-6 (IL-6), in the culture medium were detected using ELISA Kits according to the manufacturer’s instructions (Shanghai Xitang Biotechnology Co., Ltd., Shanghai, China). A Thermo microplate reader was used for the measurement of optical density (OD) at 450 nm.

### Western blot

Treated cells were collected for centrifugation, after RIPA lysis buffer containing PMSF was added. After centrifugation, the supernatants were collected for protein measurement using the BCA method. The samples were boiled for 15 min, and the protein concentration was measured via a bicinchoninic acid protein assay kit (Bio-rad, China). After resolution of equal protein for each sample in SDS-PAGE gels, the proteins were then transferred to PVDF membranes. The membranes were blocked with 5% skimmed milk in TBST for 2 h, at room temperature, on a rocker; the membranes were also incubated with corresponding primary antibodies (anti-HSP90 dilution 1:1000, anti-TLR4 dilution 1:1000, anti-GAPDH dilution 1:10000, antibodies purchased from Cell Signal Technology, USA) overnight, then secondary antibodies (1:3000 HRP-conjugated anti-goat or anti-rabbit IgG) for 1 h at 37° C. An enhanced chemiluminescence detection kit was used for the visualization. The band intensities of protein expression were acquired and analysed by Image-Pro Plus 6.0 software (Media Cybernetics, Bethesda, USA) and normalized to band intensities of GAPDH. All experiments were repeated three times.

### Statistical analysis

Statistical analysis was performed using SPSS 20.0 software. The data are presented as mean ± standard deviation. The significance of differences among experimental groups was assessed by one-way analysis of variance and inter-group pairwise comparison, known as the Student-Newman Keuls method, to detect the difference for each index. Results were considered statistically significant when the *P*-value was < 0.05.

## Supplementary Material

Supplementary Table 1

Supplementary Table 2

Supplementary Table 3

Supplementary Table 4

Supplementary Table 5
