# Supplementary material for: Investigating the mechanisms of Modified Xiaoyaosan (tiaogan-liqi prescription) in suppressing the progression of atherosclerosis, by means of integrative pharmacology and experimental validation
Source: Aging (Albany NY). 2021 Apr 4;13(8):11411–32. doi: 10.18632/aging.202832 (PMC8109114; doi:10.18632/aging.202832)
Supplement: Supplementary Table 1 [file aging-13-202832-s001.docx]

**Supplementary Table 1. Chemical Components of TGLQ.**

| **No.** | **Chemical Components of TGLQ** |
| --- | --- |
| 1 | (+)-Eudesma-4(15),7(11)-Dien-8-One |
| 2 | (1R,2R,4R)-1,7,7-Trimethylbicyclo[2.2.1]Heptan-2-Ol,Isoborneol,L-Isoborneol |
| 3 | (1R,9S)-4,11,11-Trimethyl-8-Methylenebicyclo[7.2.0]Undec-4-Ene,Caryophellene,Î’-Caryophyllene |
| 4 | (1S,2S)-2-Isopropenyl-4-Isopropylidene-1-Methyl-1-Vinylcyclohexane,Î“-Elemene |
| 5 | (3R,5S)-3,5-Dihydroxy-1-(4-Hydroxy-3,5-Dimethoxyphenyl)-7-(4-Hydroxy-3-Methoxyphenyl)Heptane |
| 6 | (3R,5S)-3-Acetoxy-5-Hydroxy-1,7-Bis(4-Hydroxy-3-Methoxyphenyl)Heptane |
| 7 | (3S,10S)-3-Isopropyl-6,10-Dimethylcyclodec-6-Ene-1,4-Dione,Curdione |
| 8 | (3S,5S)-3,5-Diacetoxy-1,7-Bis(4-Hydroxy-3-Methoxyphenyl)Heptane |
| 9 | (5S)-5-Acetoxy-1,7-Bis(4-Hydroxy-3-Methoxyphenyl)Heptan-3-One |
| 10 | (6R)-3,7,7-Trimethyl-11-Methylidenespiro[5.5]Undec-2-Ene,Î’-Chamigrene |
| 11 | (E)-1-[2,4-Dihydroxy-3-(3-Methyl-2-Butenyl)Phenyl]-3-(2,2-Dimethyl-8-Hydroxy-2H-Benzopyran-6-Yl)-2-Propen-1-One |
| 12 | (E)-1-[2,4-Dihydroxy-3-(3-Methyl-2-Butenyl)Phenyl]-3-(4-Hydroxy-3-[3-Methyl-2-Butenyl)Phenyl]-2-Propen-1-One |
| 13 | (E)-8Î’,17-Epoxylabd-12-Ene-15,16-Dial,Aframodial |
| 14 | (E)-Citral,3,7-Dimethylocta-2,6-Dienal,Citral-B |
| 15 | (R)-P-Menth-1-En-4-Ol,Terpinen-4-Ol |
| 16 | (S)-1-Methyl-4-(6-Methylhepta-1,5-Dien-2-Yl)Cyclohex-1-Ene,Î’-Bisabolene |
| 17 | (S)-5,7-Dihydroxy-2-Phenylchroman-4-One,Pinocembrin |
| 18 | [(S)-Carvone,Carvone,Thymol](http://www.tcmip.cn/TCMIP/index.php/Home/Index/cf_details.html?id=89) |
| 19 | (Trans-Trans)Farnesol,3,7,11-Trimethyldodeca-2,6,10-Trien-1-Ol,Farnesol |
| 20 | (Z)-3,7-Dimethyl-1,3,6-Octatriene ,Î’-Cis-Ocimene |
| 21 | (Z')-3,8-Dihydro-6,6',7,3'Î‘-Diligustilide |
| 22 | (Z)-4,5-Dihydro-6,7-Cis-Dihydroxy-3-Butylidene Phthalide |
| 23 | (Z)-4,5-Dihydro-6,7-Trans-Dihydroxy-3-Butylidene Phthalide |
| 24 | (Z)-6,7-Epoxy-6,7-Dihydroligustilide |
| 25 | (Z,Z')-Diligustilide |
| 26 | (Z,Z)-Î‘-Farnesene |
| 27 | [10]-Gingediol |
| 28 | [4]-Gingerol |
| 29 | [6',6''-Dimethylpyrano-(2',3':7,8)]-4'-Methoxy-3-Arylcoumarin |
| 30 | [6]-Gingediacetate |
| 31 | [6]-Gingediol |
| 32 | [6]-Gingerdione (Enol Form) |
| 33 | [6]-Gingerol |
| 34 | [8]-Gingediol |
| 35 | 1-(1,5-Dimethyl-4-Hexenyl)-4-Methyl Benzene,Î‘-Curcumene |
| 36 | 1-(3,4-Dihydroxyphenyl)-6,7-Dihydroxy-1,2-Dihydronaphthalene-2,3-Dicarboxylic Acid |
| 37 | 1-(4-Hydroxy-3-Methoxyphenyl)-3,5-Diacetoxyoctane |
| 38 | 1-(4-Hydroxy-3-Methoxyphenyl)-3,5-Octane-Diol |
| 39 | 1-(4-Hydroxy-3-Methoxyphenyl)Propan-1,2-Diol |
| 40 | 1,1,5-Trimethyl-2-Formyl-Cyclohexa-2,5-Diene-4-One |
| 41 | 1,2,3,4,6-Pentagalloylglucose |
| 42 | 1,2,3-Tri-O-Galloyl-Î’-D-Glucose |
| 43 | 1,2,4,6-Tetra-O-Galloyl-Î’-D-Glucose |
| 44 | 1,2,6-Tri-O-Galloyl-Î’-D-Glucose |
| 45 | 1,3,3-Trimethyltricyclo[2.2.1.02,6]Heptane |
| 46 | 1,3,6-Trigalloyl-Î’-D-Glucose |
| 47 | 1,3-Bis-[2-(3,4-Dihydroxyphenyl)-1-Carboxy]Ethoxycarbonyl-2-(3,4-Dihydroxyphenyl)-7,8-Dihydroxy-1,2-Dihydronaphthalene |
| 48 | 1,3-Bis-[2-(3,4-Dihydroxyphenyl)-1-Methoxycarbonyl]Ethoxycarbonyl-2-(3,4-Dihydroxyphenyl)-7,8-Dihydroxy-1,2-Dihydronaphthalene |
| 49 | 1,5-Epoxy-3-Hydroxy-1-(4-Hydroxy-3,5-Dimethoxyphenyl)-7-(4-Hydroxy-3-Methoxyphenyl)Heptane |
| 50 | 1,8-Cineole,Eucalyptol |
| 51 | 1,8-Dimethyl-4-(1-Methylenyl)-Spiro(4,5) Dec-7-Ene,Î’-Acoradiene |
| 52 | 10-Dehydrogingerdione |
| 53 | 10-Gingerol |
| 54 | 12-(Î‘-Methyl Butyryl)-14-Acetyl-2E,8E,10E-Atractylentriol |
| 55 | 12-(Î‘-Methyl Butyryl)-14-Acetyl-2E,8Z,10E-Atractylentriol |
| 56 | 13-Methyl Tetradecanoic Acid |
| 57 | 14(Î‘-Methyl Butyryl)-2E,8E,10E-Atractylentriol |
| 58 | 14(Î‘-Methyl Butyryl)-2E,8Z,10E-Atractylentriol |
| 59 | [16Î‘,23,28,30-Tetrahydroxyolean-11,13(18)-Dien-3Î’-Yl-Î’-D-Glucopyranosyl-(1â†’3)-Î’-D-Fucopyranoside](http://www.tcmip.cn/TCMIP/index.php/Home/Index/cf_details.html?id=1160) |
| 60 | 1-Isopropyl-4-Methylcyclohexa-1,4-Diene,Terpinolene,Î“-Terpinene |
| 61 | 1-Methyl-4-(Prop-1-En-2-Yl)Cyclohex-1-Ene,Limonene,Dipentene,Î‘-Limonene |
| 62 | 1-O-Galloyl-Glucose |
| 63 | 1-Tetradecanol |
| 64 | 2-(1-Oxopentyl)-Benzoic Acid Methyl Ester |
| 65 | 2,3-Dicresol |
| 66 | 2,3-O-(S)-Hexahydroxydiphenoyl-D-Glucopyranose |
| 67 | 2,4,4'-Trihydroxychalcone |
| 68 | 2,4,6-Trimethylbenzaldehyde |
| 69 | 2',4'-Dihydroxyacetophenone |
| 70 | [22-Stigmasterol](http://www.tcmip.cn/TCMIP/index.php/Home/Index/cf_details.html?id=1159) |
| 71 | 25-Hydroxy-3-Epidehydrotumulosic Acid |
| 72 | 2-Caraneol |
| 73 | 2-Heptanol |
| 74 | [2-Heptenic Acid](http://www.tcmip.cn/TCMIP/index.php/Home/Index/cf_details.html?id=1127) |
| 75 | 2-Hexanol |
| 76 | 2-Methoxy-4-(3-Methoxy-1-Propenyl)-Phenol |
| 77 | [2-Methylcyclopentanone](http://www.tcmip.cn/TCMIP/index.php/Home/Index/cf_details.html?id=1133) |
| 78 | 2-Methyl-Dodecane-5-One |
| 79 | [2-Nonenoic Acid](http://www.tcmip.cn/TCMIP/index.php/Home/Index/cf_details.html?id=1137) |
| 80 | [2-Octenic Acid](http://www.tcmip.cn/TCMIP/index.php/Home/Index/cf_details.html?id=1139) |
| 81 | 3'-(Î“,Î“-Dimethylallyl)-Kievitone |
| 82 | 3(S)-3-Butyl-4,5-Dihydrophthalide |
| 83 | 3,3'-Dimethylquercetin |
| 84 | 3,4,5-Trihydroxybenzoic Acid,Gallic Acid,Gallicacidmonohydrate |
| 85 | 3,4-Dihydroxybenzoic Acid,Protocatechuic Acid |
| 86 | 3,5-Diacetoxy-1-(4-Hydroxy-3,5-Dimethoxyphenyl)-7-(4-Hydroxy-3-Methoxyphenyl) Heptane |
| 87 | 3',7-Dihydroxy-4',6-Dimethoxyisoflavone |
| 88 | 3-Butylidene-7-Hydroxyphalide |
| 89 | 3-Butylidene-Phalide |
| 90 | 3-Butylidenephthalide |
| 91 | 3-Butyl-Phthalide |
| 92 | 3-Caraneol |
| 93 | 3-Epidehydrotumulosic Acid |
| 94 | 3-Hydroxycoumarin,Folic Acid |
| 95 | 3-Hydroxyglabrol |
| 96 | 3Î’-Hydroxy-16Î‘-Acetoxy-Lanosta-7,9(11),24-Trien-21-Oic Acid |
| 97 | 3Î’-Hydroxylanosta-7,9(11),24-Trien-21-Oic Acid |
| 98 | 3Î’-P-Hydroxybenzoyldehydrotumulosic Acid |
| 99 | 3-Isopropyl-6-Methylenecyclohex-1-Ene,3-Methylene-6-(1-Methylethyl)-Cyclohexene,Î’-Phellandrene |
| 100 | 3'-Methoxyglabridin |
| 101 | 3-Methyl-6,7,8-Trihydropyrrolo[1,2-A]Pyrimidin-2-One |
| 102 | 3-N-Butyl-3-Hydroxy-4,5,6,7-Tetrahydro-6,7-Dihydroxy Phthalide |
| 103 | 3-O-[Î’-D-Glucuronopyranosyl-(1â†’2)-O-Î’-D-Glucuronopyranosyl]-24-Hydroxyglabrolide |
| 104 | 3-O-Acetyl-Glycyrrhetinic Acid |
| 105 | 3-Octanol |
| 106 | 3-O-Galloyl Quinic Acid |
| 107 | [3-O-trans ferulylquinic acid](http://www.tcmip.cn/TCMIP/index.php/Home/Index/cf_details.html?id=17) |
| 108 | 3-Pentanol |
| 109 | 4,7-Dihydroxy-3-Butylphthalide |
| 110 | 4-Ethenyl-2,2,4-Trimethyl-3-(1-Methylethenyl)-Cyclohexane-Methanol |
| 111 | 4-Ethylresorcinol |
| 112 | [4-Hydroxy-3-Methoxy Styrene,Styrene](http://www.tcmip.cn/TCMIP/index.php/Home/Index/cf_details.html?id=203) |
| 113 | 4-Hydroxycoumarin,Folinic Acid |
| 114 | 4-Hydroxyonchocarpin |
| 115 | 4-Methyl-1-(1-Methylethyl)-3-Cyclohexen-1-Ol-Acetate |
| 116 | 4-O-Galloyl Quinic Acid |
| 117 | 4'-O-Methylglabridin |
| 118 | 5,6,7,8-Tetrahydro-2,4-Dimethylquinoline |
| 119 | 5,6,7,8-Tetrahydro-4-Methylquinoline |
| 120 | 5,7,4'-Trihydroxy-6,8,3'-Trimethoxyflavone,Labiatenic Acid,Rosmarinic Acid |
| 121 | 5-Desgalloylstachyurin |
| 122 | 5-Hydroxy-1-(3,4-Dihydroxy-5-Methoxyphenyl)-7-(4-Hydroxy-3-Methoxyphenyl)Heptan-3-One |
| 123 | 5-Hydroxy-1-(4-Hydroxy-3-Methoxyphenyl)-7-(3,4-Dihydroxy-5-Methoxyphenyl)Heptan-3-One |
| 124 | 5-Hydroxy-1-(4-Hydroxy-3-Methoxyphenyl)-7-(3,4-Dihydroxyphenyl)Heptan-3-One |
| 125 | [5-Hydroxycoumarin,Guaiacol](http://www.tcmip.cn/TCMIP/index.php/Home/Index/cf_details.html?id=1125) |
| 126 | 5-Hydroxymethyl-6-Endo-(3'-Methoxy-4'-Hydroxyphenyl)-8-Oxa-Bicyclo[3.2.1]-Oct-3-En-2-One |
| 127 | 6,6-Dimethyl-2-Methylenebicyclo[3.1.1]Heptane,Î’-Pinene |
| 128 | 6,8-Bis(C-Î’-Glucosyl)-Apigenin,Vicenin-2 |
| 129 | 6-Acetyl Gingerol |
| 130 | 6-Dehydrogingerdione |
| 131 | 6-Gingerdione |
| 132 | 6-Methoxy-7-Hydroxycoumarin |
| 133 | 6-Methylgingediacetate |
| 134 | 6-Methylgingediol |
| 135 | 6-Paradol |
| 136 | 6-Prenylated Eriodictyol |
| 137 | 6-Shogaol |
| 138 | 7-Hydroxycoumarin,Skimmetin,Umbelliferone |
| 139 | [7-Methyl-3-Methyleneocta-1,6-Diene,Myrcene,Î’-Myrcene](http://www.tcmip.cn/TCMIP/index.php/Home/Index/cf_details.html?id=142) |
| 140 | 8-Gingerol |
| 141 | 8Î’-Ethoxy Atractylenolide Iii |
| 142 | 8-Prenylated Eriodictyol |
| 143 | Acetylborneol,Bornyl Acetate,L-Bornyl Acetate |
| 144 | Adenine |
| 145 | [Adonitol](http://www.tcmip.cn/TCMIP/index.php/Home/Index/cf_details.html?id=1122) |
| 146 | Agarospirol,Hinesol |
| 147 | Albiflorin |
| 148 | Albiflorin R1 |
| 149 | Alexandrin,Daucosterol,Caproic Acid,Eleutheroside A,Sitogluside,Strumaroside,Î’-Sitosterol-Î’-D-Glucoside |
| 150 | Alexandrin,Daucosterol,Eleutheroside A |
| 151 | Alloaromadendrene |
| 152 | Alloocimene |
| 153 | [Alpha-Humulene,Humulene,Î‘-Humulene](http://www.tcmip.cn/TCMIP/index.php/Home/Index/cf_details.html?id=38) |
| 154 | Aminobutyl Canavalmine |
| 155 | Angelicide |
| 156 | [Angelicin,Isopsoralen](http://www.tcmip.cn/TCMIP/index.php/Home/Index/cf_details.html?id=619) |
| 157 | Anisic Acid |
| 158 | Asparagine |
| 159 | Atractylenolide I |
| 160 | Atractylenolide III |
| 161 | Atractylentriol |
| 162 | Atractylone |
| 163 | Azelaic Acid |
| 164 | Benzoic Acid |
| 165 | Benzoylpaeoniflorin |
| 166 | Beta-Elemene,Î’-Elemene |
| 167 | Beta-Eudesmol |
| 168 | [Bicyclo[3.1.1]Hept-2-Ene-2-Methanol, 6,6-Dimethyl,Myrtenol](http://www.tcmip.cn/TCMIP/index.php/Home/Index/cf_details.html?id=1135) |
| 169 | Bicycloelemene |
| 170 | Bisdemethoxycurcumin |
| 171 | Borneol |
| 172 | Brefeldin A |
| 173 | Butylphthalide |
| 174 | [Caffeic Acid](http://www.tcmip.cn/TCMIP/index.php/Home/Index/cf_details.html?id=97) |
| 175 | Camphor |
| 176 | Camphoric Acid |
| 177 | Canavalia Gibberellin I |
| 178 | Canavalia Gibberellin Ii |
| 179 | [Caproic Acid](http://www.tcmip.cn/TCMIP/index.php/Home/Index/cf_details.html?id=839) |
| 180 | [Caprylic Acid](http://www.tcmip.cn/TCMIP/index.php/Home/Index/cf_details.html?id=347) |
| 181 | Carene-3 |
| 182 | Carvacrol |
| 183 | Casuarictin |
| 184 | Casuariin |
| 185 | Catechin |
| 186 | Cedrol,Eudesmol,Î‘-Cedrol |
| 187 | [Cetylic Acid,Hexadecanoic Acid,Palmitic Acid](http://www.tcmip.cn/TCMIP/index.php/Home/Index/cf_details.html?id=258) |
| 188 | Choerospondin |
| 189 | Choline |
| 190 | Choline Chloride |
| 191 | Chrysophanic Acid |
| 192 | Chrysophanol |
| 193 | Chuanxiongol |
| 194 | Chuanxiongterpene |
| 195 | Chuanxiongzine |
| 196 | [Cis-9,Cis-12-Linoleic Acid,Inositol,Linoleic,Linoleic Acid](http://www.tcmip.cn/TCMIP/index.php/Home/Index/cf_details.html?id=126) |
| 197 | Citral,Neral |
| 198 | Citronellol |
| 199 | Citronellol,Menthol |
| 200 | Citronellyl Acetate |
| 201 | Cnidilide |
| 202 | Cnidium Lactone |
| 203 | Copaene |
| 204 | Corylifolinin,Isobavachalcone |
| 205 | Curcumin |
| 206 | D-8-Acetoxycarvotanacetone |
| 207 | D-Camphene |
| 208 | Decyl Acetate |
| 209 | Dehydroabietic Acid Methyl Ester |
| 210 | Dehydroeburicoic Acid |
| 211 | Dehydrotumulosic Acid |
| 212 | Dibutyl Uralsaponin A Ester |
| 213 | Diethyl Sulfide |
| 214 | Dilinoyl Palmitoyl Glyceride,Glycerol1-Monopalmitate |
| 215 | Dimethyl Azelate |
| 216 | Dimethyl Camphorate |
| 217 | Dimethyl Phthalate |
| 218 | Dimethyl Sebacate |
| 219 | D-Isoborneol |
| 220 | [D-Limonene,Limonene,Dipentene,Î‘-Phellandrene](http://www.tcmip.cn/TCMIP/index.php/Home/Index/cf_details.html?id=815) |
| 221 | Dodecanol |
| 222 | Dodecenoic Acid |
| 223 | Eburicoic Acid |
| 224 | Elemol |
| 225 | [Enanthic Acid](http://www.tcmip.cn/TCMIP/index.php/Home/Index/cf_details.html?id=1123) |
| 226 | E-Nerolidol |
| 227 | Ergosterol |
| 228 | Erythrinin C |
| 229 | Ethyl Acetate |
| 230 | Ethyl Geranate,Geranyl Acetate |
| 231 | Ethyl Heptadecanoate |
| 232 | [Ethyl Palmitate](http://www.tcmip.cn/TCMIP/index.php/Home/Index/cf_details.html?id=150) |
| 233 | Ethylisoheptadecanoate |
| 234 | Ethylisooctadecanoate |
| 235 | Ethylisopropyl Sulfide |
| 236 | Ethyl-N-Buthy-Uralsaponin A Esters |
| 237 | Ethyloctadecanoate |
| 238 | Ethylpentadecanoate |
| 239 | Ethylpropionate |
| 240 | Eugeniin |
| 241 | Eugenol Methyl Ether,Methyl Isoeugenol,Methyleugenol |
| 242 | [Eugenol,Guaiacol](http://www.tcmip.cn/TCMIP/index.php/Home/Index/cf_details.html?id=1124) |
| 243 | Fenchyl Alcohol |
| 244 | Formononetin |
| 245 | Formononetin-7-Glucoside |
| 246 | Fructose |
| 247 | Furanogermenone |
| 248 | Galanolactone |
| 249 | Galloylpaeoniflorin |
| 250 | Gancaonin A |
| 251 | Gancaonin B |
| 252 | Gancaonin C |
| 253 | Gancaonin D |
| 254 | Gancaonin E |
| 255 | Gancaonin F |
| 256 | Gancaonin I |
| 257 | Gancaonin P-3'-Methylether |
| 258 | Gancaonin X |
| 259 | Gancaonin Y |
| 260 | Gancaonin Z |
| 261 | [Geraniol,Sabinene Hydrate](http://www.tcmip.cn/TCMIP/index.php/Home/Index/cf_details.html?id=850) |
| 262 | Gingerenone A |
| 263 | Gingerenone B |
| 264 | Gingerenone C |
| 265 | Glabrolide |
| 266 | Glicoricone |
| 267 | Glisoflavanone |
| 268 | Glyarallin B |
| 269 | Glycycoumarin |
| 270 | Glycyrdione D |
| 271 | Glycyrin |
| 272 | Glycyrol |
| 273 | Glycyrrhetinic Acid |
| 274 | Glycyrrhetol |
| 275 | Glycyrrhisoflavanone |
| 276 | Glycyrrhisoflavone |
| 277 | Glycyrrhiza-Flavonol A |
| 278 | Glycyrrhizic Acid |
| 279 | Glyoxal |
| 280 | Glyurallin A |
| 281 | Glyuranolide |
| 282 | Glyzaglabrin |
| 283 | Heptane |
| 284 | Hispaglabridin A |
| 285 | Hispaglabridin B |
| 286 | Homosenkyunolide H |
| 287 | Homosenkyunolide I |
| 288 | Î‘-Acoradiene |
| 289 | Î‘-Bergamotene |
| 290 | Î‘-Cedrene |
| 291 | [Î‘-Cubebene](http://www.tcmip.cn/TCMIP/index.php/Home/Index/cf_details.html?id=927) |
| 292 | Î‘-Curcumene,Î‘-Curcumene(R-) |
| 293 | [Î‘-Longipinene](http://www.tcmip.cn/TCMIP/index.php/Home/Index/cf_details.html?id=1131) |
| 294 | Î‘-Pinene |
| 295 | [Î‘-Spinasterol](http://www.tcmip.cn/TCMIP/index.php/Home/Index/cf_details.html?id=485) |
| 296 | [Î‘-Terpineol](http://www.tcmip.cn/TCMIP/index.php/Home/Index/cf_details.html?id=187) |
| 297 | Î’-Amyrin Acetate |
| 298 | Î’-Cedrene |
| 299 | Î’-Curcumene |
| 300 | Î’-Elemol |
| 301 | Î’-Farnesene |
| 302 | Î’-Fenchene |
| 303 | Î’-Humulene |
| 304 | Î’-Phellandrene |
| 305 | Î’-Santalol |
| 306 | Î“-Acoradiene |
| 307 | [Î“-Heptalactone](http://www.tcmip.cn/TCMIP/index.php/Home/Index/cf_details.html?id=1126) |
| 308 | [Î“-Octalactone](http://www.tcmip.cn/TCMIP/index.php/Home/Index/cf_details.html?id=1138) |
| 309 | [Î“-Undecalactone](http://www.tcmip.cn/TCMIP/index.php/Home/Index/cf_details.html?id=1163) |
| 310 | Î”1(7)-Menthene |
| 311 | Î”2,4-Dihydrophthalic Anhydride |
| 312 | Î”3-Menthene |
| 313 | Î”-Acoradiene |
| 314 | Isocnidilide |
| 315 | Isoeugenol |
| 316 | Isogingerenone B |
| 317 | Isoglycyrol |
| 318 | Isograbrol |
| 319 | Isolicoflavonol |
| 320 | Isoliquiritigenin |
| 321 | Isoliquiritin |
| 322 | Isomenthone |
| 323 | Isomenthone,L-Menthone |
| 324 | Isoononin |
| 325 | [Isoquercitrin,Isoquercetrin,Kuwanon H](http://www.tcmip.cn/TCMIP/index.php/Home/Index/cf_details.html?id=383) |
| 326 | Isoraifolin |
| 327 | Isoschaftoside |
| 328 | Isotrifoliol |
| 329 | Isovaleraldehyde |
| 330 | Isoviolanthin |
| 331 | Juniper Camphor |
| 332 | [Kaempferitrin](http://www.tcmip.cn/TCMIP/index.php/Home/Index/cf_details.html?id=1128) |
| 333 | Kanzonol B |
| 334 | Kanzonol K |
| 335 | Kanzonol L |
| 336 | Kumatakenin |
| 337 | Lauric Acid |
| 338 | L-Î‘-Amino-Î”-Hydroxyvaleric Acid |
| 339 | Licoagrochalcone A |
| 340 | Licoagroisoflavone |
| 341 | Licoagropin |
| 342 | Licoagroside C |
| 343 | Licoarylcoumarin |
| 344 | Licobenzofuran |
| 345 | Licobichalcone |
| 346 | Licocoumarone |
| 347 | Licoflavonal |
| 348 | Licofuranocoumarin |
| 349 | Licoisoflavone |
| 350 | Licoisoflavone B |
| 351 | Licoleafol |
| 352 | Licopyranocoumarin |
| 353 | Licoricesaponin A3 |
| 354 | Licoricesaponin B2 |
| 355 | Licoricesaponin C2 |
| 356 | Licoricesaponin D3 |
| 357 | Licoricesaponin E2 |
| 358 | Licoricesaponin F3 |
| 359 | Licoricesaponin G2 |
| 360 | Licoricesaponin H2 |
| 361 | Licoricesaponin J2 |
| 362 | Licoricesaponin K2 |
| 363 | Licoricidin |
| 364 | Licoricone |
| 365 | Licorisoflavan A |
| 366 | Licuroside |
| 367 | [Lignoceric Acid](http://www.tcmip.cn/TCMIP/index.php/Home/Index/cf_details.html?id=995) |
| 368 | Ligustilide |
| 369 | Ligustilide Dimer |
| 370 | [Linalool](http://www.tcmip.cn/TCMIP/index.php/Home/Index/cf_details.html?id=1129) |
| 371 | [Linolenic Acid](http://www.tcmip.cn/TCMIP/index.php/Home/Index/cf_details.html?id=261) |
| 372 | Liquiritigenin |
| 373 | Liquiritigenin 4'-O-Î’-D-Apio-D-Furanosyl(1â†’2)-Î’-D-Glucopyranoside |
| 374 | Liquiritigenin-7,4'-Diglucoside |
| 375 | Liquiritin |
| 376 | [Longifolene](http://www.tcmip.cn/TCMIP/index.php/Home/Index/cf_details.html?id=1130) |
| 377 | [Longispinogenin](http://www.tcmip.cn/TCMIP/index.php/Home/Index/cf_details.html?id=1132) |
| 378 | Lupiwighteone |
| 379 | Maackiain |
| 380 | Maruzen M,P-Ethylphenol |
| 381 | Menthol |
| 382 | Menthone |
| 383 | Menthyl Acetate |
| 384 | Meso-3,5-Diacetoxy-1,7-Bis-(4-Hydroxy-3-Methoxyphenyl) Heptane |
| 385 | Methyl 18Î‘-Hydroxyglycyrrhetate |
| 386 | Methyl Allyl Sulfide |
| 387 | Methyl Benzoate |
| 388 | Methyl Hexadecanate,Methyl Palmitate |
| 389 | Methyl Isobutyl Ketone |
| 390 | Methyl Linoleate |
| 391 | Methyl Pentadecanoate |
| 392 | Methyl Phenylacetate |
| 393 | Methyl-24-Hydroxy-11-Deoxoglycyrrhetate |
| 394 | Methyl-24-Hydroxyglycyrrhetate |
| 395 | Methylacetate |
| 396 | Methylglycyrrhetate |
| 397 | Methylglyoxal |
| 398 | Methyl-N-Butyl-Uralsaponin A Esters |
| 399 | M-Ethylphenol |
| 400 | Myristic Acid |
| 401 | [Myrtanol](http://www.tcmip.cn/TCMIP/index.php/Home/Index/cf_details.html?id=1134) |
| 402 | Narcissin |
| 403 | N-Butylaldehyde |
| 404 | Neocnidilide |
| 405 | Neoisoliquiritin |
| 406 | Neoliquiritin |
| 407 | Neouralenol |
| 408 | Nerol |
| 409 | Niacin,Nicotinic Acid |
| 410 | Nicotiflorin |
| 411 | N-Nonane |
| 412 | N-Nonanol |
| 413 | N-Octane |
| 414 | Nonan-2-One |
| 415 | [Nonanoic Acid](http://www.tcmip.cn/TCMIP/index.php/Home/Index/cf_details.html?id=1136) |
| 416 | [Nootkatone](http://www.tcmip.cn/TCMIP/index.php/Home/Index/cf_details.html?id=844) |
| 417 | N-Propanol |
| 418 | N-Tricosane |
| 419 | [N-Tridecane](http://www.tcmip.cn/TCMIP/index.php/Home/Index/cf_details.html?id=1162) |
| 420 | N-Valerophenone-O-Carboxylic Acid |
| 421 | O-Acetylpachymic Acid-25-Ol |
| 422 | O-Cresol |
| 423 | [Octadecanoic?Acid,Stearic Acid](http://www.tcmip.cn/TCMIP/index.php/Home/Index/cf_details.html?id=278) |
| 424 | Octanal |
| 425 | Ordoritin-Glucoside |
| 426 | Oxypaeoniflorin |
| 427 | Pachymic Acid |
| 428 | Pachymic Acid Methyl Ester |
| 429 | Paeonianiin E |
| 430 | Paeonianin A |
| 431 | Paeonianin B |
| 432 | Paeonianin C |
| 433 | Paeonianin D |
| 434 | Paeoniflorigenone |
| 435 | Paeoniflorin |
| 436 | Paeonilactone A |
| 437 | Paeonilactone B |
| 438 | Paeonilactone C |
| 439 | Paeonioflorin |
| 440 | Paeonol,Scopoletin |
| 441 | Palbinone |
| 442 | Pavilion,Scopoletin,Scopoletol,Trigonelline |
| 443 | P-Cresol |
| 444 | P-Cymol,P-Cymene,Umbelliferone |
| 445 | Pedunculagin |
| 446 | [Pentanic Acid,Scopolin](http://www.tcmip.cn/TCMIP/index.php/Home/Index/cf_details.html?id=1140) |
| 447 | Peonin |
| 448 | Perilal |
| 449 | Perlolyrine |
| 450 | [Petroselaidic Acid](http://www.tcmip.cn/TCMIP/index.php/Home/Index/cf_details.html?id=1141) |
| 451 | [Petroselinic Acid](http://www.tcmip.cn/TCMIP/index.php/Home/Index/cf_details.html?id=1142) |
| 452 | Phaseol |
| 453 | Phaseollinisoflavan |
| 454 | [Phenol](http://www.tcmip.cn/TCMIP/index.php/Home/Index/cf_details.html?id=523) |
| 455 | Phthalic Anhydride |
| 456 | Pinicolic Acid A |
| 457 | Pipecolic Acid |
| 458 | Polyporenic Acid C |
| 459 | Poricoic Acid A |
| 460 | Poricoic Acid B |
| 461 | Poricoic Acid C |
| 462 | Poricoic Acid D |
| 463 | Poricoic Acid Dm |
| 464 | Poricoic Acid G |
| 465 | Poricoic Acid H |
| 466 | Propionaldehyde |
| 467 | P-Tolyl-Methyl Carbinol Diferuloyl Methane |
| 468 | [Pulegone](http://www.tcmip.cn/TCMIP/index.php/Home/Index/cf_details.html?id=1143) |
| 469 | Pyrethrin I |
| 470 | Pyrethrin Ii |
| 471 | Retinol |
| 472 | Rutin,Rutoside,Vitamin P |
| 473 | Safrole |
| 474 | [Saikochromic Acid](http://www.tcmip.cn/TCMIP/index.php/Home/Index/cf_details.html?id=1144) |
| 475 | [Saikochromoside A](http://www.tcmip.cn/TCMIP/index.php/Home/Index/cf_details.html?id=1145) |
| 476 | [Saikogenin E](http://www.tcmip.cn/TCMIP/index.php/Home/Index/cf_details.html?id=1146) |
| 477 | [Saikogenin F](http://www.tcmip.cn/TCMIP/index.php/Home/Index/cf_details.html?id=1147) |
| 478 | [Saikogenin G](http://www.tcmip.cn/TCMIP/index.php/Home/Index/cf_details.html?id=1148) |
| 479 | [Saikosaponin A](http://www.tcmip.cn/TCMIP/index.php/Home/Index/cf_details.html?id=1149) |
| 480 | [Saikosaponin C](http://www.tcmip.cn/TCMIP/index.php/Home/Index/cf_details.html?id=1150) |
| 481 | [Saikosaponin D](http://www.tcmip.cn/TCMIP/index.php/Home/Index/cf_details.html?id=1151) |
| 482 | [Saikosaponin Q1](http://www.tcmip.cn/TCMIP/index.php/Home/Index/cf_details.html?id=1152) |
| 483 | [Saikosaponin Q2](http://www.tcmip.cn/TCMIP/index.php/Home/Index/cf_details.html?id=1153) |
| 484 | [Saikosaponin S1](http://www.tcmip.cn/TCMIP/index.php/Home/Index/cf_details.html?id=1154) |
| 485 | [Saikosaponin T](http://www.tcmip.cn/TCMIP/index.php/Home/Index/cf_details.html?id=1155) |
| 486 | [Saikosaponin V](http://www.tcmip.cn/TCMIP/index.php/Home/Index/cf_details.html?id=1156) |
| 487 | [Saikosaponin V1](http://www.tcmip.cn/TCMIP/index.php/Home/Index/cf_details.html?id=1157) |
| 488 | [Saikosaponin V2](http://www.tcmip.cn/TCMIP/index.php/Home/Index/cf_details.html?id=1158) |
| 489 | Sebacic Acid |
| 490 | Sedanonic Acid |
| 491 | Semilicoisoflavone B |
| 492 | Senkyunolide |
| 493 | Senkyunolide C |
| 494 | Senkyunolide D |
| 495 | Senkyunolide E |
| 496 | Senkyunolide F |
| 497 | Senkyunolide G |
| 498 | Senkyunolide H |
| 499 | Senkyunolide J |
| 500 | Senkyunolide K |
| 501 | Senkyunolide L |
| 502 | Senkyunolide M |
| 503 | Senkyunolide N |
| 504 | Senkyunolide Q |
| 505 | Senkyunone |
| 506 | Sigmoidin B |
| 507 | [Sitosterol,Î’-Sitosterol](http://www.tcmip.cn/TCMIP/index.php/Home/Index/cf_details.html?id=66) |
| 508 | Spathulenol |
| 509 | [Stigmasterol](http://www.tcmip.cn/TCMIP/index.php/Home/Index/cf_details.html?id=185) |
| 510 | Stigmasterol-Î’-D-Glucoside |
| 511 | Strictinin |
| 512 | Succinic Acid |
| 513 | Tellimagrandin I |
| 514 | Terpinolene,Î‘-Terpinolene |
| 515 | Thujene |
| 516 | [Thymol](http://www.tcmip.cn/TCMIP/index.php/Home/Index/cf_details.html?id=1161) |
| 517 | Trametenolic Acid |
| 518 | Trans-Beta-Ocimene |
| 519 | Tricyclene |
| 520 | Trimethylamine |
| 521 | Trimethylamine- Hcl |
| 522 | Tumulosic Acid |
| 523 | Tumulosic Acid Methyl Ester |
| 524 | Turmerone |
| 525 | Undecanoic Acid |
| 526 | Uracil |
| 527 | Uralene |
| 528 | Uralenin |
| 529 | Uralenneoside |
| 530 | Uralenol |
| 531 | Uralenol-3-Methylether |
| 532 | Uralsaponin A |
| 533 | Uralsaponin B |
| 534 | Uralstilbene |
| 535 | [Vanillin](http://www.tcmip.cn/TCMIP/index.php/Home/Index/cf_details.html?id=205) |
| 536 | [Vanillin Acetate](http://www.tcmip.cn/TCMIP/index.php/Home/Index/cf_details.html?id=1164) |
| 537 | Violanthin |
| 538 | Vitamin B12 |
| 539 | Wallichilide |
| 540 | Xambioona |
| 541 | Z-1S,5R-Î’-Pinen-10-Yl-Î’-Vicianoside |
| 542 | Z-3',8',3'Î‘,7'Î‘-Tetrahydro-6,3',7,7'Î‘-Diligustilide-8'-One |
| 543 | Zedoarondiol |
| 544 | Zingerone |
| 545 | Zingiberene |
| 546 | Zingiberol |
| 547 | Z-Nerolidol |
| 548 | Zonarene |
