# Supplementary material for: Investigating the mechanisms of Modified Xiaoyaosan (tiaogan-liqi prescription) in suppressing the progression of atherosclerosis, by means of integrative pharmacology and experimental validation
Source: Aging (Albany NY). 2021 Apr 4;13(8):11411–32. doi: 10.18632/aging.202832 (PMC8109114; doi:10.18632/aging.202832)
Supplement: Supplementary Table 2 [file aging-13-202832-s002.docx]

Supplementary Table 2. Putative targets of TGLQ.

| **No.** | **Gene symbol** | **No.** | **Gene symbol** | **No.** | **Gene symbol** |
| --- | --- | --- | --- | --- | --- |
| 1 | glk | 324 | GABRG3 | 647 | SCN3A |
| 2 | TOP2A | 325 | ams | 648 | NAGA |
| 3 | OTC | 326 | fabF | 649 | SCN3B |
| 4 | choB | 327 | fabH | 650 | SREBF1 |
| 5 | nplT | 328 | fabI | 651 | LTB4R |
| 6 | TM0268 | 329 | amy | 652 | SCN5A |
| 7 | cenC | 330 | GLTP | 653 | HDAC9 |
| 8 | PLA2G1B | 331 | SIGMAR1 | 654 | glpF |
| 9 | ALDH18A1 | 332 | COMT | 655 | SCN7A |
| 10 | GLUL | 333 | xynA | 656 | HSD17B11 |
| 11 | LSS | 334 | SQLE | 657 | MAN1B1 |
| 12 | LCT | 335 | 72 | 658 | SCN9A |
| 13 | INS | 336 | dhbE | 659 | CYP27B1 |
| 14 | C8G | 337 | GLRA3 | 660 | glcB |
| 15 | gdhB | 338 | celC307 | 661 | CYP19A1 |
| 16 | CPQ | 339 | ganB | 662 | FADS1 |
| 17 | pptA | 340 | fabZ | 663 | FADS2 |
| 18 | ARG1 | 341 | ARF1 | 664 | MTTP |
| 19 | lpxC | 342 | GUCA1A | 665 | MED1 |
| 20 | ARG2 | 343 | tvaI | 666 | SCN11A |
| 21 | SHBG | 344 | tvaII | 667 | ADH1B |
| 22 | 9 | 345 | ARF6 | 668 | mdlB |
| 23 | EIF3F | 346 | budC | 669 | MAOB |
| 24 | GRIN3A | 347 | entB | 670 | fucI |
| 25 | GRIN3B | 348 | PPARA | 671 | bglA |
| 26 | E | 349 | AADAT | 672 | ITPR1 |
| 27 | cumD | 350 | xynY | 673 | RXRB |
| 28 | GPT2 | 351 | PPARD | 674 | ubiC |
| 29 | IL1B | 352 | PPARG | 675 | mutL |
| 30 | ksi | 353 | lgtC | 676 | eryF |
| 31 | cocE | 354 | PPP1CC | 677 | FFAR1 |
| 32 | RORA | 355 | GRIN2A | 678 | RXRG |
| 33 | RCVRN | 356 | GRIN2B | 679 | ACOX1 |
| 34 | lacZ | 357 | ECI2 | 680 | argG |
| 35 | VDR | 358 | GRIN2C | 681 | LTB4R2 |
| 36 | MIF | 359 | GRIN2D | 682 | fadH |
| 37 | PVR | 360 | cutA | 683 | P4HA1 |
| 38 | GRIK1 | 361 | PRKCA | 684 | HSD17B6 |
| 39 | GRIK2 | 362 | PRKCB | 685 | CYTH2 |
| 40 | GRIK3 | 363 | ATP5A1 | 686 | P4HA2 |

| **No.** | **Gene symbol** | **No.** | **Gene symbol** | **No.** | **Gene symbol** |
| --- | --- | --- | --- | --- | --- |
| 41 | GRIK4 | 364 | ESRRA | 687 | PLD1 |
| 42 | GRIK5 | 365 | ESRRB | 688 | PLD2 |
| 43 | AMY1A | 366 | stx2eB | 689 | MMACHC |
| 44 | GCK | 367 | Rv1372 | 690 | SUCNR1 |
| 45 | SLC25A15 | 368 | TM0024 | 691 | PLOD1 |
| 46 | SLC25A18 | 369 | ccrA | 692 | TMLHE |
| 47 | SLC15A1 | 370 | CDK6 | 693 | SLC13A1 |
| 48 | CASR | 371 | cobT | 694 | PLOD3 |
| 49 | BEVgp1 | 372 | GM2A | 695 | SDHA |
| 50 | LIP3 | 373 | hutH | 696 | RDH5 |
| 51 | ilvE | 374 | GRIN1 | 697 | SLC13A2 |
| 52 | ttgR | 375 | IFNB1 | 698 | SDHB |
| 53 | CYP2C8 | 376 | ALB | 699 | SDHC |
| 54 | LCN2 | 377 | NCAN | 700 | SLC13A3 |
| 55 | cypC | 378 | DAO | 701 | ptr2 |
| 56 | SFTPD | 379 | AHR | 702 | SDHD |
| 57 | SLC25A2 | 380 | NADSYN1 | 703 | MMAA |
| 58 | ADH5 | 381 | aroQ | 704 | RDH8 |
| 59 | nrdB | 382 | mutY | 705 | atpE |
| 60 | SLC7A11 | 383 | EARS2 | 706 | MMAB |
| 61 | HSD11B1 | 384 | xly | 707 | SRD5A2 |
| 62 | HSD11B2 | 385 | nfsB | 708 | NQO1 |
| 63 | CTSB | 386 | gag-pol | 709 | FOLR1 |
| 64 | cat3 | 387 | oxyR | 710 | AOX1 |
| 65 | NPPB | 388 | cat | 711 | FOLR2 |
| 66 | HNF4A | 389 | SULT2A1 | 712 | FOLR3 |
| 67 | ncsA | 390 | FPGS | 713 | HBB |
| 68 | JAK1 | 391 | cex | 714 | BBOX1 |
| 69 | HNF4G | 392 | dmsA | 715 | HBA1 |
| 70 | TYR | 393 | HDAC2 | 716 | UL2 |
| 71 | DHFRL1 | 394 | SLC1A1 | 717 | ribH |
| 72 | AASS | 395 | SLC1A2 | 718 | Moth_1208 |
| 73 | TAT | 396 | SLC1A3 | 719 | OXCT1 |
| 74 | CLEC4E | 397 | SLC1A6 | 720 | linB |
| 75 | SULT2B1 | 398 | OAZ1 | 721 | folA |
| 76 | HSP90AA1 | 399 | ODC1 | 722 | OXCT2 |
| 77 | FURIN | 400 | SLC1A7 | 723 | hemB |
| 78 | TM1468 | 401 | OAZ2 | 724 | ACHE |
| 79 | PPT1 | 402 | OAZ3 | 725 | UCKL1 |
| 80 | SLCO1B3 | 403 | ATP5B | 726 | ACOT13 |

| **No.** | **Gene symbol** | **No.** | **Gene symbol** | **No.** | **Gene symbol** |
| --- | --- | --- | --- | --- | --- |
| 81 | NAGS | 404 | luxF | 727 | PTGIR |
| 82 | AKT1 | 405 | tonB | 728 | PTGIS |
| 83 | TXNRD1 | 406 | pobA | 729 | DHODH |
| 84 | PYGL | 407 | CSNK2A1 | 730 | CYP2A6 |
| 85 | PYGM | 408 | luxS | 731 | ALDH1L1 |
| 86 | SMOX | 409 | SEC14L2 | 732 | pdp |
| 87 | cyp102A1 | 410 | SMARCA5 | 733 | pyrDA |
| 88 | ctxB | 411 | lipB | 734 | glyA |
| 89 | AADACL2 | 412 | PPP3CA | 735 | metH |
| 90 | spoII | 413 | S100B | 736 | pyrC |
| 91 | ADRB1 | 414 | HOXA10 | 737 | dmg |
| 92 | ADRB2 | 415 | PRDX5 | 738 | pyrD |
| 93 | YWHAE | 416 | NR1I2 | 739 | pyrE |
| 94 | HSPA2 | 417 | ABL1 | 740 | rsuA |
| 95 | gag | 418 | NR1I3 | 741 | TYMS |
| 96 | NCOA1 | 419 | MBL2 | 742 | PNP |
| 97 | FTCD | 420 | S100P | 743 | trxB |
| 98 | NCOA2 | 421 | ALOX5 | 744 | UL30 |
| 99 | NFKB1 | 422 | nahD | 745 | phr |
| 100 | NFKB2 | 423 | BAAT | 746 | MUT |
| 101 | RAB9A | 424 | NME1 | 747 | MTHFD1 |
| 102 | PSAT1 | 425 | camC | 748 | QPRT |
| 103 | CA1 | 426 | RNASE1 | 749 | MTHFD2 |
| 104 | PLA2G2A | 427 | COX6A2 | 750 | dapD |
| 105 | CA2 | 428 | BGLAP | 751 | FAAH |
| 106 | LTF | 429 | ASPH | 752 | ALDH1A1 |
| 107 | CA3 | 430 | MT-CO1 | 753 | ATIC |
| 108 | CA4 | 431 | dps | 754 | ALDH1A2 |
| 109 | PLA2G2D | 432 | MT-CO2 | 755 | ALDH1A3 |
| 110 | PLA2G2E | 433 | MT-CO3 | 756 | ALAD |
| 111 | CA6 | 434 | ALAS1 | 757 | HCAR2 |
| 112 | GABRA1 | 435 | ALAS2 | 758 | P2RY12 |
| 113 | CA7 | 436 | SLC7A8 | 759 | AMN |
| 114 | GABRA2 | 437 | ACY1 | 760 | HCAR3 |
| 115 | GABRA3 | 438 | SPTLC1 | 761 | MTHFR |
| 116 | CA9 | 439 | SERPINC1 | 762 | DHRS4L1 |
| 117 | ABAT | 440 | SPTLC2 | 763 | arnA |
| 118 | GABRA4 | 441 | ACY3 | 764 | LRAT |
| 119 | LPL | 442 | UBC | 765 | AMT |
| 120 | GABRA5 | 443 | GSS | 766 | PHOSPHO1 |

| **No.** | **Gene symbol** | **No.** | **Gene symbol** | **No.** | **Gene symbol** |
| --- | --- | --- | --- | --- | --- |
| 121 | GABRA6 | 444 | pol | 767 | ung |
| 122 | OAT | 445 | shp | 768 | DPYD |
| 123 | xylA | 446 | GLRA1 | 769 | P3H1 |
| 124 | GLUD1 | 447 | KYNU | 770 | P3H2 |
| 125 | fadR | 448 | GLRA2 | 771 | MPN348 |
| 126 | GLUD2 | 449 | IARS | 772 | P3H3 |
| 127 | PRKACA | 450 | CANT1 | 773 | SUCLA2 |
| 128 | gag-pro-pol | 451 | VC1968 | 774 | RETSAT |
| 129 | MB | 452 | pufC | 775 | DHRS3 |
| 130 | PPA_RS05235 | 453 | atpH | 776 | DHRS4 |
| 131 | botB | 454 | VEGFA | 777 | nos |
| 132 | galM | 455 | puhA | 778 | NNMT |
| 133 | CYP1B1 | 456 | AARS2 | 779 | fgs |
| 134 | OPLAH | 457 | IARS2 | 780 | thyA |
| 135 | GNPDA1 | 458 | GPR18 | 781 | RDH11 |
| 136 | AR | 459 | SRR | 782 | PCYT1A |
| 137 | GIG18 | 460 | AARS | 783 | BLLF1 |
| 138 | ATP5C1 | 461 | YARS2 | 784 | RDH12 |
| 139 | NOS2 | 462 | CAMP | 785 | PCYT1B |
| 140 | nef | 463 | ASRGL1 | 786 | RDH13 |
| 141 | RXRA | 464 | GCSH | 787 | CUBN |
| 142 | NOS3 | 465 | CLPP | 788 | RDH14 |
| 143 | SIGLEC1 | 466 | pufL | 789 | ACO2 |
| 144 | PVgp1 | 467 | env | 790 | APOD |
| 145 | clcA | 468 | pufM | 791 | TCN1 |
| 146 | UBA1 | 469 | COX7A1 | 792 | deoA |
| 147 | stxB | 470 | CACNA1C | 793 | MTFMT |
| 148 | malE | 471 | CBS | 794 | XDH |
| 149 | GAD1 | 472 | CACNA1D | 795 | gadA |
| 150 | GAD2 | 473 | GLDC | 796 | SUCLG1 |
| 151 | DR_A0149 | 474 | spg | 797 | SUCLG2 |
| 152 | GRID1 | 475 | CACNA1F | 798 | RBP1 |
| 153 | GRID2 | 476 | MGMT | 799 | sdsA1 |
| 154 | PGR | 477 | AGXT2 | 800 | RLBP1 |
| 155 | malP | 478 | COX5A | 801 | RBP3 |
| 156 | AMY2A | 479 | narG | 802 | aspC |
| 157 | AMY2B | 480 | COX5B | 803 | TTHA0718 |
| 158 | GMPS | 481 | narH | 804 | TK |
| 159 | malT | 482 | F2 | 805 | gcvT |
| 160 | GATB | 483 | narI | 806 | SLC25A10 |
| **No.** | **Gene symbol** | **No.** | **Gene symbol** | **No.** | **Gene symbol** |
| 161 | SLC25A22 | 484 | GLYATL1 | 807 | polA |
| 162 | FOLH1 | 485 | GARS | 808 | casp8 |
| 163 | GPT | 486 | GLYATL2 | 809 | aldA |
| 164 | GLS | 487 | xynZ | 810 | CES1 |
| 165 | ESR1 | 488 | CACNA1S | 811 | mvaA |
| 166 | ESR2 | 489 | COX7B | 812 | NFKBIA |
| 167 | GATM | 490 | COX7C | 813 | ESRRG |
| 168 | ABO | 491 | ADSS | 814 | ITGAL |
| 169 | TLR4 | 492 | METAP2 | 815 | PECR |
| 170 | moxC | 493 | oppA | 816 | ACP1 |
| 171 | AMD1 | 494 | LYZ | 817 | cbh |
| 172 | PIM1 | 495 | ASS1 | 818 | GPBAR1 |
| 173 | alsB | 496 | SLC36A1 | 819 | ruvB |
| 174 | tesA | 497 | SLC19A3 | 820 | EFTUD1 |
| 175 | lecB | 498 | cc4 | 821 | acrB |
| 176 | PRLR | 499 | CTSD | 822 | ppcA |
| 177 | AKR1C1 | 500 | ACADSB | 823 | FECH |
| 178 | AKR1C2 | 501 | TRPA1 | 824 | ITGB2 |
| 179 | ENPEP | 502 | NARS | 825 | APRT |
| 180 | ASNS | 503 | PAH | 826 | mtnN |
| 181 | AKR1C3 | 504 | CARS2 | 827 | SRPK2 |
| 182 | TNF | 505 | GSTP1 | 828 | ptd |
| 183 | CBR1 | 506 | PTH | 829 | DKFZp686P18130 |
| 184 | tcp14 | 507 | DARS2 | 830 | FABP6 |
| 185 | cgt | 508 | B2M | 831 | MTAP |
| 186 | SLC7A1 | 509 | cyp158a2 | 832 | ctaC |
| 187 | SLC7A2 | 510 | COX6B1 | 833 | ADH1C |
| 188 | SF3B3 | 511 | SLC32A1 | 834 | ctaD |
| 189 | SLC7A3 | 512 | VARS | 835 | ACACB |
| 190 | SLC7A4 | 513 | SERPINB3 | 836 | HMGCR |
| 191 | STK17B | 514 | TRPV1 | 837 | CFTR |
| 192 | PMP2 | 515 | pcaG | 838 | xyn10C |
| 193 | potD | 516 | GNMT | 839 | YARS |
| 194 | potF | 517 | pcaH | 840 | xyl |
| 195 | amyA | 518 | TRPV3 | 841 | TOP2B |
| 196 | amyE | 519 | CRH | 842 | APP |
| 197 | BCAT1 | 520 | CAD | 843 | xynC |
| 198 | GPER1 | 521 | CTPS1 | 844 | ALOX15 |
| 199 | BCAT2 | 522 | SGR_RS28980 | 845 | GNAS |
| 200 | HSD3B1 | 523 | S100G | 846 | IGKV2-30 |

| **No.** | **Gene symbol** | **No.** | **Gene symbol** | **No.** | **Gene symbol** |
| --- | --- | --- | --- | --- | --- |
| 201 | amyM | 524 | GLRB | 847 | PIK3R1 |
| 202 | aacC1 | 525 | rub | 848 | xlnA |
| 203 | amyP | 526 | PPAT | 849 | GAPDHS |
| 204 | pab | 527 | PTPN1 | 850 | cslA |
| 205 | treZ | 528 | NEIL1 | 851 | CLEC14A |
| 206 | NQO2 | 529 | pac | 852 | KHSRP |
| 207 | GRM1 | 530 | CACNB1 | 853 | ITGB3 |
| 208 | pyp | 531 | MT-ND1 | 854 | PIK3CA |
| 209 | GOT1 | 532 | CTH | 855 | MTNR1A |
| 210 | GRM4 | 533 | CACNB2 | 856 | MTNR1B |
| 211 | GOT2 | 534 | F10 | 857 | SLC5A2 |
| 212 | ATP1A1 | 535 | CACNB3 | 858 | rbsB |
| 213 | GRM7 | 536 | PIN1 | 859 | rbsD |
| 214 | ATP1A2 | 537 | CACNB4 | 860 | ADCY2 |
| 215 | GRM8 | 538 | CDO1 | 861 | PLK1 |
| 216 | ATP1A3 | 539 | RABGGTA | 862 | ADCY5 |
| 217 | GLS2 | 540 | PCCB | 863 | PI4K2B |
| 218 | IL6 | 541 | nlpI | 864 | snca |
| 219 | PIK3CG | 542 | RABGGTB | 865 | ebgA |
| 220 | nprS | 543 | hld | 866 | abfA |
| 221 | chqB | 544 | AGXT | 867 | KANSL3 |
| 222 | gfo | 545 | SLC1A4 | 868 | SLC2A1 |
| 223 | celA | 546 | SLC1A5 | 869 | ITGA5 |
| 224 | celB | 547 | ACCS | 870 | PARS2 |
| 225 | celD | 548 | HI_1317 | 871 | SLC6A14 |
| 226 | celA1 | 549 | fdhF | 872 | atpF |
| 227 | GABRB1 | 550 | CSAD | 873 | menC |
| 228 | GABRB2 | 551 | Ethr | 874 | PYCR1 |
| 229 | GABRB3 | 552 | DARS | 875 | PYCR2 |
| 230 | LALBA | 553 | ASS | 876 | ABCC5 |
| 231 | PTGS1 | 554 | DPP4 | 877 | DBI |
| 232 | PTGS2 | 555 | MTRR | 878 | PROSC |
| 233 | HRSP12 | 556 | COX4I1 | 879 | PYCRL |
| 234 | celS | 557 | snoaL | 880 | purF |
| 235 | GCLC | 558 | pcp | 881 | PRODH |
| 236 | cel5A | 559 | GCAT | 882 | TUBB |
| 237 | ANXA1 | 560 | SLC6A5 | 883 | ddh |
| 238 | GCLM | 561 | SDS | 884 | PPIA |
| 239 | CTRB1 | 562 | murI | 885 | PPIB |
| 240 | CASP3 | 563 | asd | 886 | PPIC |
| 241 | RUVBL2 | 564 | sufS | 887 | PPIF |

| **No.** | **Gene symbol** | **No.** | **Gene symbol** | **No.** | **Gene symbol** |
| --- | --- | --- | --- | --- | --- |
| 242 | FKBP1A | 565 | PIPOX | 888 | PPIG |
| 243 | ansB | 566 | SLC6A9 | 889 | PPIH |
| 244 | pvdQ | 567 | FARS2 | 890 | murE |
| 245 | TRAPPC3 | 568 | est | 891 | tyrB |
| 246 | engF | 569 | COX6C | 892 | P5CR2 |
| 247 | PKIA | 570 | NARS2 | 893 | SLC6A7 |
| 248 | metE | 571 | SARS | 894 | GLO1 |
| 249 | lamB | 572 | NFS1 | 895 | argB |
| 250 | HIBCH | 573 | mndD | 896 | L3HYPDH |
| 251 | ACTB | 574 | CARS | 897 | CYP1A2 |
| 252 | fhuA | 575 | OPRK1 | 898 | actIII |
| 253 | SYK | 576 | TRPM8 | 899 | SRD5A3 |
| 254 | nedA | 577 | TH | 900 | CACNA1A |
| 255 | eco | 578 | pgl | 901 | CACNA1B |
| 256 | ALDH2 | 579 | PAICS | 902 | CACNA1G |
| 257 | CCBL2 | 580 | SLC38A3 | 903 | CACNA1H |
| 258 | SMS | 581 | BHMT | 904 | CACNA1I |
| 259 | GRIA1 | 582 | COX8A | 905 | CACNG1 |
| 260 | GRIA2 | 583 | ADSSL1 | 906 | GART |
| 261 | CA12 | 584 | PHYKPL | 907 | folP |
| 262 | GRIA3 | 585 | fmt | 908 | CACNA2D1 |
| 263 | GRIA4 | 586 | IGHG1 | 909 | CYP2E1 |
| 264 | CA14 | 587 | ulaG | 910 | CACNA2D2 |
| 265 | UGT3A1 | 588 | IL4I1 | 911 | CYP2C9 |
| 266 | CA5A | 589 | gshA | 912 | CACNA2D3 |
| 267 | CA5B | 590 | bioB | 913 | CYP3A43 |
| 268 | PTK2B | 591 | BHMT2 | 914 | KRT2 |
| 269 | GGCX | 592 | lytA | 915 | CYP3A4 |
| 270 | eltB | 593 | MTR | 916 | CYP3A5 |
| 271 | IGHG2 | 594 | GLYAT | 917 | CYP3A7 |
| 272 | AURKB | 595 | CCBL1 | 918 | purN |
| 273 | BAMF_RS28815 | 596 | CPA1 | 919 | GSTA1 |
| 274 | NR3C1 | 597 | DCN | 920 | IGKC |
| 275 | NR3C2 | 598 | adh | 921 | CYP11B2 |
| 276 | fusA | 599 | SHMT1 | 922 | kcsA |
| 277 | NR0B1 | 600 | FARSA | 923 | NR1H2 |
| 278 | HSD17B1 | 601 | SLC25A12 | 924 | NR1H3 |
| 279 | cslB | 602 | SHMT2 | 925 | catA |
| 280 | EPRS | 603 | FARSB | 926 | bphC |
| 281 | tetX | 604 | SLC25A13 | 927 | MAPK8IP1 |
| 282 | GABRD | 605 | HNF1A | 928 | CYP2D6 |

| **No.** | **Gene symbol** | **No.** | **Gene symbol** | **No.** | **Gene symbol** |
| --- | --- | --- | --- | --- | --- |
| 283 | GABRE | 606 | HLA-B | 929 | KRT12 |
| 284 | CEBPB | 607 | pflB | 930 | DHFR |
| 285 | HK1 | 608 | SCN2A | 931 | FEN1 |
| 286 | LCTL | 609 | SERPINA1 | 932 | POLB |
| 287 | PFAS | 610 | SCN2B | 933 | NPY |
| 288 | GABRP | 611 | BCHE | 934 | PRSS1 |
| 289 | GABRQ | 612 | fabG3 | 935 | CYB5A |
| 290 | cpo | 613 | dctD | 936 | dhaK |
| 291 | HCK | 614 | SCN4A | 937 | FTH1 |
| 292 | repA | 615 | SCN4B | 938 | HARS |
| 293 | KCNMA1 | 616 | AKR1D1 | 939 | AHSP |
| 294 | LY96 | 617 | ATP2A1 | 940 | HDC |
| 295 | PPP3R1 | 618 | SRD5A1 | 941 | SOD2 |
| 296 | CSNK2B | 619 | HPGDS | 942 | dadX |
| 297 | aspA | 620 | eno | 943 | nikA |
| 298 | ispD | 621 | ELOVL4 | 944 | talB |
| 299 | CALM1 | 622 | SCN8A | 945 | EPOR |
| 300 | GLT6D1 | 623 | SLC8A1 | 946 | lysA |
| 301 | DNMT1 | 624 | TGFBR2 | 947 | FXN |
| 302 | aglA | 625 | ORM1 | 948 | dapA |
| 303 | KRTAP5-2 | 626 | OGDH | 949 | AZIN2 |
| 304 | mglB | 627 | TRDMT1 | 950 | IYD |
| 305 | KRTAP5-3 | 628 | SCN10A | 951 | HDAC8 |
| 306 | NR1H4 | 629 | hisD | 952 | NEIL2 |
| 307 | LGALS2 | 630 | bfr | 953 | GAMT |
| 308 | LGALS3 | 631 | ISYNA1 | 954 | ASL |
| 309 | LGALS7 | 632 | PAPSS1 | 955 | TFRC |
| 310 | LGSN | 633 | HAO1 | 956 | HAL |
| 311 | celCCF | 634 | onr | 957 | NOS1 |
| 312 | celCCG | 635 | ALDH5A1 | 958 | TF |
| 313 | VLDLR | 636 | mexA | 959 | DRD1 |
| 314 | DNPEP | 637 | rhaA | 960 | DRD2 |
| 315 | PAEP | 638 | CYP17A1 | 961 | DRD3 |
| 316 | ppgmk | 639 | CNR1 | 962 | DRD4 |
| 317 | NUDT9 | 640 | SOAT1 | 963 | DRD5 |
| 318 | GABRG1 | 641 | CNR2 | 964 | KARS |
| 319 | RHO | 642 | SOAT2 | 965 | CKM |
| 320 | GABRG2 | 643 | SCN1A | 966 | tyrS |
| 321 | fabB | 644 | HA | 967 | CP |
| 322 | cbhA | 645 | SCN1B | 968 | EGLN1 |
| 323 | ACSL4 | 646 | ACSL3 |  |  |
