# Supplementary material for: Investigating the mechanisms of Modified Xiaoyaosan (tiaogan-liqi prescription) in suppressing the progression of atherosclerosis, by means of integrative pharmacology and experimental validation
Source: Aging (Albany NY). 2021 Apr 4;13(8):11411–32. doi: 10.18632/aging.202832 (PMC8109114; doi:10.18632/aging.202832)
Supplement: Supplementary Table 3 [file aging-13-202832-s003.docx]

Supplementary Table 3. Therapeutic targets of atherosclerosis

| **No.** | **Atherosclerosis** | **No.** | **Atherosclerosis** | **No.** | **Atherosclerosis** |
| --- | --- | --- | --- | --- | --- |
| 1 | ABCA1 | 336 | SELPLG | 671 | SKP2 |
| 2 | ABCG5 | 337 | SORL1 | 672 | SLC3A2 |
| 3 | ADIPOQ | 338 | SRI | 673 | SLC9A1 |
| 4 | AGER | 339 | SREBF1 | 674 | SLC12A3 |
| 5 | AHR | 340 | SYK | 675 | SLC22A1 |
| 6 | ALOX5 | 341 | TAGLN | 676 | SNAI2 |
| 7 | ALOX5AP | 342 | TCF21 | 677 | SMARCA4 |
| 8 | APOA4 | 343 | TFPI | 678 | SMPD1 |
| 9 | BCL3 | 344 | TIE1 | 679 | SOS1 |
| 10 | BMP2 | 345 | TLR3 | 680 | SP1 |
| 11 | CCL3 | 346 | TNFRSF1A | 681 | SPARC |
| 12 | CCL4 | 347 | TSC1 | 682 | SRY |
| 13 | CNR2 | 348 | TUFM | 683 | SSRP1 |
| 14 | CYP27A1 | 349 | UCP1 | 684 | SSTR2 |
| 15 | IL18 | 350 | ZFP36 | 685 | ST14 |
| 16 | SELP | 351 | SOCS1 | 686 | STAT6 |
| 17 | CAV1 | 352 | IRS2 | 687 | TAC1 |
| 18 | HMGB1 | 353 | BECN1 | 688 | TFAM |
| 19 | F2 | 354 | MBTPS1 | 689 | NR2F2 |
| 20 | CLU | 355 | TNFSF12 | 690 | TGFB1I1 |
| 21 | CASR | 356 | CREG1 | 691 | THBS4 |
| 22 | MMP2 | 357 | HDAC3 | 692 | THM |
| 23 | LMNA | 358 | LDB2 | 693 | THOP1 |
| 24 | LDLRAP1 | 359 | SLC33A1 | 694 | TP73 |
| 25 | ENG | 360 | IL32 | 695 | CRISP2 |
| 26 | WRN | 361 | AIMP1 | 696 | TRAF1 |
| 27 | SLC17A4 | 362 | ADAMTS3 | 697 | TRAF2 |
| 28 | SCARB1 | 363 | CCL4L2 | 698 | TRAF6 |
| 29 | CX3CR1 | 364 | CLOCK | 699 | TRPC6 |
| 30 | ACE | 365 | NOS1AP | 700 | TTR |
| 31 | DECR1 | 366 | ZMPSTE24 | 701 | UMOD |
| 32 | EDN1 | 367 | CXCR6 | 702 | VCL |
| 33 | FABP4 | 368 | NISCH | 703 | VIM |
| 34 | FN1 | 369 | MMRN1 | 704 | XBP1 |
| 35 | HMOX1 | 370 | DKK1 | 705 | ZNF148 |
| 36 | IGF1 | 371 | KCNH4 | 706 | ZNF202 |
| 37 | IL10 | 372 | ABCA12 | 707 | LRP8 |
| 38 | IL17A | 373 | PTPN22 | 708 | NPHS2 |
| 39 | LCAT | 374 | SGSM3 | 709 | SCG2 |
| 40 | MMP9 | 375 | TOR2A | 710 | FGF23 |

| **No.** | **Atherosclerosis** | **No.** | **Atherosclerosis** | **No.** | **Atherosclerosis** |
| --- | --- | --- | --- | --- | --- |
| 41 | NFKB1 | 376 | RMC1 | 711 | KMT2D |
| 42 | OLR1 | 377 | IL20 | 712 | TAM |
| 43 | PLTP | 378 | FOXP3 | 713 | SOAT2 |
| 44 | PPARA | 379 | TMED7 | 714 | PLPP3 |
| 45 | PTX3 | 380 | TNFRSF12A | 715 | AOC3 |
| 46 | REN | 381 | IL17D | 716 | PDE5A |
| 47 | TLR2 | 382 | SAMD9 | 717 | DGAT1 |
| 48 | TP53 | 383 | MYDGF | 718 | TNFSF13 |
| 49 | CXCR4 | 384 | HRASLS | 719 | TNFSF9 |
| 50 | RETN | 385 | AS3MT | 720 | ADAM9 |
| 51 | PCSK9 | 386 | NCEH1 | 721 | RIPK2 |
| 52 | MIR155 | 387 | SEMA6A | 722 | TNFRSF11A |
| 53 | CCR2 | 388 | NIF3L1 | 723 | SUCLA2 |
| 54 | IL1A | 389 | ADAM33 | 724 | NRP1 |
| 55 | NFE2L2 | 390 | KISS1R | 725 | SQSTM1 |
| 56 | PON2 | 391 | DNER | 726 | KALRN |
| 57 | SPP1 | 392 | EARS2 | 727 | CH25H |
| 58 | TXN | 393 | OSCAR | 728 | TBX18 |
| 59 | APOM | 394 | OSCP1 | 729 | SLC16A3 |
| 60 | NLRP3 | 395 | KCNH8 | 730 | HGS |
| 61 | TNFRSF11B | 396 | KLF14 | 731 | TMSB10 |
| 62 | UCP2 | 397 | SGMS2 | 732 | IL1RL1 |
| 63 | VWF | 398 | KIF6 | 733 | ARHGEF2 |
| 64 | LIPG | 399 | IL27 | 734 | GPR55 |
| 65 | ATM | 400 | TICAM2 | 735 | CD163 |
| 66 | CCR5 | 401 | SERPINA13P | 736 | SLIT2 |
| 67 | MMP3 | 402 | CCL4L1 | 737 | PPIG |
| 68 | NPY | 403 | MIRLET7G | 738 | FADS2 |
| 69 | PIK3CG | 404 | MIR126 | 739 | ABCG2 |
| 70 | TCF7L2 | 405 | MIR143 | 740 | GSTO1 |
| 71 | CDKN2B-AS1 | 406 | MIR144 | 741 | IL27RA |
| 72 | AKR1B1 | 407 | MIR19A | 742 | ADAMTS1 |
| 73 | APOH | 408 | MIR210 | 743 | PTGES |
| 74 | CDKN2A | 409 | MIR24-1 | 744 | H6PD |
| 75 | EGR1 | 410 | MIR27A | 745 | GOSR2 |
| 76 | ESR2 | 411 | MIR27B | 746 | ECE2 |
| 77 | F7 | 412 | MIR29A | 747 | KMT2B |
| 78 | MMP12 | 413 | MIR30C1 | 748 | PCLAF |
| 79 | PECAM1 | 414 | MIR30C2 | 749 | MTSS1 |
| 80 | CXCL12 | 415 | TNFSF12-TNFSF13 | 750 | HS3ST1 |

| **No.** | **Atherosclerosis** | **No.** | **Atherosclerosis** | **No.** | **Atherosclerosis** |
| --- | --- | --- | --- | --- | --- |
| 81 | TGFB1 | 416 | TMED7-TICAM2 | 751 | KCNE2 |
| 82 | THBS1 | 417 | CBSL | 752 | HDAC5 |
| 83 | ACE2 | 418 | ABR | 753 | PARP3 |
| 84 | APOA5 | 419 | ADAM8 | 754 | ABCB6 |
| 85 | ALB | 420 | ADAM10 | 755 | IL18BP |
| 86 | ANGPT1 | 421 | ADCY8 | 756 | EBI3 |
| 87 | CD44 | 422 | ADRA2B | 757 | LPCAT3 |
| 88 | CSF2 | 423 | ACAN | 758 | MPZL2 |
| 89 | DPP4 | 424 | APLNR | 759 | LYPLA1 |
| 90 | ELN | 425 | AHCY | 760 | MERTK |
| 91 | EPRS | 426 | AKT2 | 761 | FST |
| 92 | GJA4 | 427 | ALDH2 | 762 | CREB3 |
| 93 | GNB3 | 428 | AMBP | 763 | SEMA4D |
| 94 | HIF1A | 429 | ANPEP | 764 | KAT5 |
| 95 | TNC | 430 | ANXA2 | 765 | SSSCA1 |
| 96 | LTA | 431 | AOC2 | 766 | CXCL13 |
| 97 | MOK | 432 | APEX1 | 767 | POSTN |
| 98 | CCL5 | 433 | APOC2 | 768 | CAMKK2 |
| 99 | SOAT1 | 434 | RHOA | 769 | KHDRBS1 |
| 100 | SREBF2 | 435 | STS | 770 | LBX1 |
| 101 | STAT1 | 436 | ATOH1 | 771 | DCTN6 |
| 102 | TIMP1 | 437 | ATP7A | 772 | EBP |
| 103 | TNFSF4 | 438 | ATR | 773 | OGA |
| 104 | TFPI2 | 439 | BCL2 | 774 | NFAT5 |
| 105 | APOL1 | 440 | GSTM1 | 775 | TRAF3IP2 |
| 106 | XPR1 | 441 | BCR | 776 | NES |
| 107 | NOX4 | 442 | BDNF | 777 | CYSLTR1 |
| 108 | FTO | 443 | BGN | 778 | CCR9 |
| 109 | IL33 | 444 | BLM | 779 | NOXA1 |
| 110 | MIR21 | 445 | BMP7 | 780 | FRS2 |
| 111 | ACAT1 | 446 | BRCA1 | 781 | PPARGC1A |
| 112 | ADM | 447 | KLF5 | 782 | COPS5 |
| 113 | ANGPT2 | 448 | BTF3P11 | 783 | TMED1 |
| 114 | BSG | 449 | C1QBP | 784 | RIPK3 |
| 115 | CAMP | 450 | DDR1 | 785 | KERA |
| 116 | CHIT1 | 451 | CALU | 786 | ADAMTS5 |
| 117 | CCR7 | 452 | CAPG | 787 | SLC2A6 |
| 118 | EPHX2 | 453 | CAPN1 | 788 | SNF8 |
| 119 | FCGR3A | 454 | CAST | 789 | CARD8 |
| 120 | FLT1 | 455 | CASP9 | 790 | KLRK1 |

| **No.** | **Atherosclerosis** | **No.** | **Atherosclerosis** | **No.** | **Atherosclerosis** |
| --- | --- | --- | --- | --- | --- |
| 121 | GABPA | 456 | CCNA2 | 791 | SCAP |
| 122 | GPX1 | 457 | CD5L | 792 | PALLD |
| 123 | CXCL1 | 458 | CD28 | 793 | MPRIP |
| 124 | INSR | 459 | CD74 | 794 | MCF2L |
| 125 | ITGB2 | 460 | CDC42 | 795 | SASH1 |
| 126 | MMP8 | 461 | CDH13 | 796 | SIRT3 |
| 127 | MTTP | 462 | CDK5 | 797 | ANGPTL2 |
| 128 | PAPPA | 463 | CDK9 | 798 | TRAM1 |
| 129 | PGF | 464 | CDKN1A | 799 | CES3 |
| 130 | PLAU | 465 | CDKN1C | 800 | SLC16A8 |
| 131 | MAPK1 | 466 | CTSC | 801 | DDAH2 |
| 132 | MAPK3 | 467 | CCR6 | 802 | PADI4 |
| 133 | PTGIR | 468 | CNP | 803 | DDAH1 |
| 134 | SORT1 | 469 | CNR1 | 804 | CARHSP1 |
| 135 | S100A8 | 470 | COL15A1 | 805 | NUP62 |
| 136 | S100A12 | 471 | COMP | 806 | ZNF318 |
| 137 | CX3CL1 | 472 | CR1 | 807 | POU2F3 |
| 138 | THBD | 473 | CRY1 | 808 | SOSTDC1 |
| 139 | TM7SF2 | 474 | CRYGD | 809 | ADGRA2 |
| 140 | VLDLR | 475 | CSF1R | 810 | APPL1 |
| 141 | PLA2G6 | 476 | CTF1 | 811 | ARHGEF26 |
| 142 | TNFSF10 | 477 | CTSL | 812 | TES |
| 143 | HSPB3 | 478 | CTSS | 813 | SLC17A5 |
| 144 | KLF4 | 479 | CYLD | 814 | PCOLCE2 |
| 145 | KL | 480 | CYP2C8 | 815 | GREM1 |
| 146 | NR1H3 | 481 | CYP2D6 | 816 | HBP1 |
| 147 | UTS2 | 482 | CYP2E1 | 817 | PALD1 |
| 148 | ADAMTS7 | 483 | CYP2J2 | 818 | SLC39A1 |
| 149 | IL37 | 484 | CYP3A4 | 819 | IL17B |
| 150 | F11R | 485 | CYP7A1 | 820 | EIF3K |
| 151 | ANGPTL4 | 486 | CYP26A1 | 821 | IGKV2D-29 |
| 152 | CHDH | 487 | DAG1 | 822 | KLF15 |
| 153 | NR0B1 | 488 | DAP | 823 | SETD2 |
| 154 | AIF1 | 489 | BRINP1 | 824 | PYCARD |
| 155 | AKT1 | 490 | DCN | 825 | CD274 |
| 156 | ALOX15B | 491 | DEFB4A | 826 | ICOS |
| 157 | APP | 492 | DHCR7 | 827 | NPC1L1 |
| 158 | APRT | 493 | NQO1 | 828 | HILPDA |
| 159 | ARG1 | 494 | DNAH8 | 829 | PADI1 |
| 160 | VPS51 | 495 | DNASE1 | 830 | IL19 |

| **No.** | **Atherosclerosis** | **No.** | **Atherosclerosis** | **No.** | **Atherosclerosis** |
| --- | --- | --- | --- | --- | --- |
| 161 | CASP1 | 496 | ATN1 | 831 | SOST |
| 162 | CASP3 | 497 | RCAN1 | 832 | MLXIPL |
| 163 | CBS | 498 | DSPP | 833 | ADIPOR1 |
| 164 | CD34 | 499 | TYMP | 834 | IRAK4 |
| 165 | CD59 | 500 | EDA | 835 | DCTN4 |
| 166 | CD68 | 501 | S1PR1 | 836 | GP6 |
| 167 | CES1 | 502 | EFNB1 | 837 | TLR7 |
| 168 | CRK | 503 | EGFR | 838 | ZC3HC1 |
| 169 | MAPK14 | 504 | ETS1 | 839 | TM6SF2 |
| 170 | CST3 | 505 | EVPL | 840 | PTOV1 |
| 171 | CTSB | 506 | F2RL1 | 841 | P2RY13 |
| 172 | CTSK | 507 | PTK2B | 842 | TLR9 |
| 173 | CYBB | 508 | FBN2 | 843 | TREM1 |
| 174 | DNMT1 | 509 | FGF1 | 844 | SOX18 |
| 175 | ELK1 | 510 | FGF13 | 845 | DLL4 |
| 176 | F5 | 511 | FGFR1 | 846 | TET2 |
| 177 | FCGR3B | 512 | FLNA | 847 | DYM |
| 178 | FGF2 | 513 | FMOD | 848 | DPP8 |
| 179 | FOXO1 | 514 | FOS | 849 | CDKAL1 |
| 180 | FMO3 | 515 | FPR2 | 850 | TESC |
| 181 | GAS6 | 516 | FSHMD1A | 851 | ATG16L1 |
| 182 | GC | 517 | NR5A2 | 852 | BATF3 |
| 183 | GOT2 | 518 | GCK | 853 | KMT2E |
| 184 | CFH | 519 | NR6A1 | 854 | APOBR |
| 185 | HLA-C | 520 | GJA5 | 855 | LTB4R2 |
| 186 | HMGCR | 521 | GLP1R | 856 | CYP26B1 |
| 187 | NR4A1 | 522 | GPI | 857 | SLC2A9 |
| 188 | HSD11B1 | 523 | GPX4 | 858 | SUCNR1 |
| 189 | HSPA4 | 524 | GRN | 859 | SLC2A4RG |
| 190 | HSPB1 | 525 | NR3C1 | 860 | ACKR3 |
| 191 | HSPB2 | 526 | GSR | 861 | TBX20 |
| 192 | ID3 | 527 | GSTM2 | 862 | PNPLA2 |
| 193 | IGF1R | 528 | GTF2H1 | 863 | CD248 |
| 194 | IL1RN | 529 | GTF3A | 864 | CD177 |
| 195 | IL15 | 530 | HAL | 865 | AHRR |
| 196 | TNFRSF9 | 531 | HAS2 | 866 | MAGEE1 |
| 197 | INS | 532 | SERPIND1 | 867 | CCAR2 |
| 198 | IRAK1 | 533 | HDLBP | 868 | GAS5 |
| 199 | MMP13 | 534 | HMGA1 | 869 | SLC39A8 |
| 200 | MT2A | 535 | HSPA2 | 870 | NOD2 |

| **No.** | **Atherosclerosis** | **No.** | **Atherosclerosis** | **No.** | **Atherosclerosis** |
| --- | --- | --- | --- | --- | --- |
| 201 | COX2 | 536 | HTN3 | 871 | ROBO3 |
| 202 | P2RY2 | 537 | IDH2 | 872 | ARHGEF28 |
| 203 | PIK3CA | 538 | IFI27 | 873 | PDIA2 |
| 204 | PIK3CB | 539 | IGFBP7 | 874 | P2RY12 |
| 205 | PIK3CD | 540 | IKBKB | 875 | IL25 |
| 206 | PTGDS | 541 | IL2 | 876 | KCTD15 |
| 207 | RYR3 | 542 | IL5 | 877 | ELOVL6 |
| 208 | S100A9 | 543 | IL6R | 878 | NOX5 |
| 209 | SAA@ | 544 | IL7 | 879 | ADIPOR2 |
| 210 | SCD | 545 | IL9 | 880 | DHX40 |
| 211 | SFTPD | 546 | IL11 | 881 | STEAP4 |
| 212 | ADAM17 | 547 | ILK | 882 | EHMT1 |
| 213 | HNF1A | 548 | IDO1 | 883 | PDGFD |
| 214 | TERT | 549 | INPP5D | 884 | SP6 |
| 215 | TIMP3 | 550 | IRF5 | 885 | PNPLA3 |
| 216 | TNFRSF4 | 551 | ISG20 | 886 | APOL6 |
| 217 | VTN | 552 | ITGAD | 887 | COLEC12 |
| 218 | WNT5A | 553 | ITGAM | 888 | ARHGAP24 |
| 219 | XDH | 554 | ITGB4 | 889 | SESN2 |
| 220 | CNBP | 555 | ITIH4 | 890 | SPZ1 |
| 221 | AIMP2 | 556 | JAK2 | 891 | RETNLB |
| 222 | NR4A3 | 557 | JUP | 892 | ORAI1 |
| 223 | TNFSF11 | 558 | KCNA3 | 893 | HSH2D |
| 224 | ADAM15 | 559 | KIT | 894 | TSLP |
| 225 | NR1I2 | 560 | LCN2 | 895 | SAMD1 |
| 226 | GRAP2 | 561 | LEPR | 896 | DPP9 |
| 227 | NTN1 | 562 | LIPA | 897 | MYOCD |
| 228 | ADAMTS4 | 563 | LMNB1 | 898 | ORMDL3 |
| 229 | GDF15 | 564 | LOX | 899 | PRAP1 |
| 230 | HDAC9 | 565 | LRP5 | 900 | TMEM18 |
| 231 | NR1H4 | 566 | LTB | 901 | SLCO6A1 |
| 232 | NAMPT | 567 | LTBR | 902 | GPR119 |
| 233 | AHSA1 | 568 | LYZ | 903 | HT |
| 234 | HPSE | 569 | SMAD3 | 904 | IL23R |
| 235 | CAPN10 | 570 | SMAD7 | 905 | PDIK1L |
| 236 | RNF19A | 571 | MAF | 906 | MARCH10 |
| 237 | POLDIP2 | 572 | MAOA | 907 | PAQR7 |
| 238 | NOX1 | 573 | MAZ | 908 | PRSS55 |
| 239 | HPGDS | 574 | MC4R | 909 | UNC5B |
| 240 | ANGPTL3 | 575 | CD46 | 910 | COPD |

| **No.** | **Atherosclerosis** | **No.** | **Atherosclerosis** | **No.** | **Atherosclerosis** |
| --- | --- | --- | --- | --- | --- |
| 241 | TRIB3 | 576 | MCS | 911 | HCAR2 |
| 242 | VKORC1 | 577 | MDM2 | 912 | ENPP7 |
| 243 | OR10A4 | 578 | MEF2C | 913 | NANOS2 |
| 244 | MIR145 | 579 | MAP3K5 | 914 | NANOS3 |
| 245 | MIR221 | 580 | MFGE8 | 915 | FCRL6 |
| 246 | PGR-AS1 | 581 | CXCL9 | 916 | IRGM |
| 247 | ABCA4 | 582 | MAP3K10 | 917 | IRF2BP2 |
| 248 | ADD1 | 583 | KMT2A | 918 | GSTK1 |
| 249 | AGTR2 | 584 | MMP7 | 919 | TBPL2 |
| 250 | ANXA5 | 585 | MMP11 | 920 | HACD4 |
| 251 | APOBEC1 | 586 | MPG | 921 | CIMT |
| 252 | AQP1 | 587 | MPV17 | 922 | MIR122 |
| 253 | AR | 588 | MTAP | 923 | MIR127 |
| 254 | BMP4 | 589 | COX1 | 924 | MIR130A |
| 255 | CAD | 590 | ND5 | 925 | MIR146A |
| 256 | CAT | 591 | ND6 | 926 | MIR152 |
| 257 | CDKN1B | 592 | RNR2 | 927 | MIR185 |
| 258 | CEL | 593 | MTRR | 928 | MIR19B1 |
| 259 | CCR3 | 594 | MYH9 | 929 | MIR216A |
| 260 | LTB4R | 595 | MYLK | 930 | MIR222 |
| 261 | CP | 596 | NCF2 | 931 | MIR223 |
| 262 | CREB1 | 597 | NDUFA2 | 932 | MIR224 |
| 263 | CSF1 | 598 | NFE2 | 933 | MIR302A |
| 264 | CYP1A1 | 599 | NFKB2 | 934 | MIR34A |
| 265 | CYP1A2 | 600 | NME2 | 935 | MIR135B |
| 266 | CYP2B6 | 601 | NOS1 | 936 | MIR378A |
| 267 | HBEGF | 602 | NOV | 937 | MIR497 |
| 268 | DUSP1 | 603 | PNP | 938 | MIR499A |
| 269 | EGF | 604 | NPPA | 939 | ECSCR |
| 270 | EPHA2 | 605 | NPPB | 940 | NCF1 |
| 271 | EPHB2 | 606 | NRF1 | 941 | NME1-NME2 |
| 272 | ESRRB | 607 | NT5E | 942 | ZGLP1 |
| 273 | F2R | 608 | DDR2 | 943 | MIR876 |
| 274 | FABP5 | 609 | OSBP | 944 | C20orf181 |
| 275 | ACSL1 | 610 | P4HB | 945 | HLP |
| 276 | FGB | 611 | PEBP1 | 946 | DEFB4B |
| 277 | GCH1 | 612 | FURIN | 947 | MIR664A |
| 278 | GH1 | 613 | PCSK6 | 948 | MICA |
| 279 | GHSR | 614 | PAEP | 949 | KLRC4-KLRK1 |
| 280 | GJA1 | 615 | PAK1 | 950 | LINC-ROR |

| **No.** | **Atherosclerosis** | **No.** | **Atherosclerosis** | **No.** | **Atherosclerosis** |
| --- | --- | --- | --- | --- | --- |
| 281 | GPER1 | 616 | PCDH8 | 951 | TP53COR1 |
| 282 | GSK3B | 617 | PCNA | 952 | SIRT1-AS |
| 283 | GSTT1 | 618 | PCOLCE | 953 | HP |
| 284 | GZMB | 619 | PCSK5 | 954 | HRH1 |
| 285 | HABP2 | 620 | PDE4A | 955 | AGT |
| 286 | HGF | 621 | PDGFA | 956 | HSPA1B |
| 287 | NRG1 | 622 | PDK4 | 957 | ALOX15 |
| 288 | HNF4A | 623 | SLC26A4 | 958 | IFNG |
| 289 | HSPA1A | 624 | SERPINB9 | 959 | APOE |
| 290 | HSP90AA1 | 625 | PIN1 | 960 | IGF2 |
| 291 | HSPG2 | 626 | PLIN1 | 961 | BMP8A |
| 292 | IFNA1 | 627 | POU2F1 | 962 | CCL2 |
| 293 | IFNA13 | 628 | PPP3CA | 963 | LTA4H |
| 294 | IGFBP1 | 629 | PREP | 964 | CETP |
| 295 | CYR61 | 630 | PRF1 | 965 | MMP1 |
| 296 | IL2RA | 631 | PRKAA1 | 966 | CRP |
| 297 | CXCR2 | 632 | PRKAA2 | 967 | CYBA |
| 298 | IRF1 | 633 | PRKAB1 | 968 | ESR1 |
| 299 | IRF6 | 634 | PRKCB | 969 | FASLG |
| 300 | ITGAV | 635 | MAPK7 | 970 | NOS2 |
| 301 | ITGAX | 636 | MAP2K1 | 971 | PARP1 |
| 302 | ITGB3 | 637 | PRL | 972 | ICAM1 |
| 303 | KDR | 638 | PROS1 | 973 | PLAT |
| 304 | KISS1 | 639 | PROX1 | 974 | IL6 |
| 305 | KNG1 | 640 | LGMN | 975 | LDLR |
| 306 | LGALS3 | 641 | RELN | 976 | LPA |
| 307 | LIPE | 642 | PSMC5 | 977 | PPARG |
| 308 | LRP6 | 643 | PSMD9 | 978 | NECTIN2 |
| 309 | MEFV | 644 | PTGER3 | 979 | SERPINE1 |
| 310 | MGP | 645 | PTGIS | 980 | NOS3 |
| 311 | MIF | 646 | PTHLH | 981 | NPC1 |
| 312 | NR3C2 | 647 | PTN | 982 | PLA2G2A |
| 313 | MMP14 | 648 | PTPN1 | 983 | SOCS3 |
| 314 | MSRA | 649 | PTPRF | 984 | PON1 |
| 315 | CYTB | 650 | PVR | 985 | PTGS2 |
| 316 | RNR1 | 651 | RAG1 | 986 | SOD2 |
| 317 | TRNL1 | 652 | RASGRF1 | 987 | SIRT1 |
| 318 | TRNL2 | 653 | RFX5 | 988 | SOD1 |
| 319 | MYC | 654 | RGS1 | 989 | STAT3 |
| 320 | MYD88 | 655 | RGS2 | 990 | TLR4 |

| **No.** | **Atherosclerosis** | **No.** | **Atherosclerosis** | **No.** | **Atherosclerosis** |
| --- | --- | --- | --- | --- | --- |
| 321 | NOTCH1 | 656 | ROS1 | 991 | TNF |
| 322 | NPPC | 657 | S100A1 | 992 | TRPV1 |
| 323 | PDGFB | 658 | S100B | 993 | TTPA |
| 324 | ENPP1 | 659 | SAT1 | 994 | VCAM1 |
| 325 | PDZK1 | 660 | CCL11 | 995 | VEGFA |
| 326 | PLA2G4A | 661 | CCL17 | 996 | APOA1 |
| 327 | PPARD | 662 | CCL18 | 997 | BRD4 |
| 328 | PTAFR | 663 | CCL20 | 998 | PLA2G7 |
| 329 | PTGS1 | 664 | CCL23 | 999 | LXR |
| 330 | RARRES2 | 665 | CCL25 | 1000 | APOC3 |
| 331 | RB1 | 666 | SDC1 | 1001 | SERPINB2 |
| 332 | RBP4 | 667 | SRSF1 | 1002 | ROCK1 |
| 333 | RENBP | 668 | SRSF2 | 1003 | MAPK11 |
| 334 | CCL19 | 669 | SHBG | 1004 | NR1H2 |
| 335 | CCL21 | 670 | ST3GAL4 | 1005 | IL1B |
