# Supplementary material for: Investigating the mechanisms of Modified Xiaoyaosan (tiaogan-liqi prescription) in suppressing the progression of atherosclerosis, by means of integrative pharmacology and experimental validation
Source: Aging (Albany NY). 2021 Apr 4;13(8):11411–32. doi: 10.18632/aging.202832 (PMC8109114; doi:10.18632/aging.202832)
Supplement: Supplementary Table 4 [file aging-13-202832-s004.docx]

Supplementary Table 4. Topological analysis information of the 114 common targets.

| **No.** | **name** | **Betweenness Centrality** | **Closeness Centrality** | **Degree** |
| --- | --- | --- | --- | --- |
| 1 | INS | 0.12166543 | 0.73202614 | 74 |
| 2 | IL6 | 0.08369088 | 0.71794872 | 70 |
| 3 | ALB | 0.08968958 | 0.71794872 | 69 |
| 4 | AKT1 | 0.06510883 | 0.68711656 | 63 |
| 5 | TNF | 0.03672405 | 0.64367816 | 54 |
| 6 | VEGFA | 0.04011396 | 0.64 | 52 |
| 7 | PTGS2 | 0.04323699 | 0.61538462 | 48 |
| 8 | IL1B | 0.02109584 | 0.60869565 | 45 |
| 9 | TLR4 | 0.02064134 | 0.60215054 | 42 |
| 10 | CASP3 | 0.01858105 | 0.59259259 | 40 |
| 11 | HSP90AA1 | 0.03327799 | 0.58638743 | 39 |
| 12 | ESR1 | 0.03270171 | 0.58947368 | 39 |
| 13 | PPARG | 0.0176697 | 0.58333333 | 37 |
| 14 | APP | 0.02482241 | 0.57731959 | 37 |
| 15 | NOS3 | 0.0200064 | 0.58947368 | 36 |
| 16 | NR3C1 | 0.01518658 | 0.56852792 | 34 |
| 17 | HPGDS | 0.01505022 | 0.56565657 | 33 |
| 18 | SREBF1 | 0.02479361 | 0.56281407 | 32 |
| 19 | PPIG | 0.01167935 | 0.56 | 30 |
| 20 | AR | 0.00965094 | 0.55445545 | 28 |
| 21 | CYP2E1 | 0.01032787 | 0.55445545 | 28 |
| 22 | SOD2 | 0.00899412 | 0.55445545 | 27 |
| 23 | CYP3A4 | 0.00740195 | 0.54368932 | 27 |
| 24 | AHR | 0.00269592 | 0.54368932 | 26 |
| 25 | NFKB1 | 0.00550866 | 0.53846154 | 26 |
| 26 | F2 | 0.00879139 | 0.54368932 | 25 |
| 27 | PIK3CA | 0.0090974 | 0.53080569 | 24 |
| 28 | PPARA | 0.00638535 | 0.54634146 | 24 |
| 29 | NOS2 | 0.00376398 | 0.53846154 | 23 |
| 30 | NPY | 0.00430495 | 0.52093023 | 23 |
| 31 | CNR1 | 0.0040631 | 0.52830189 | 22 |
| 32 | ALOX5 | 0.00468986 | 0.52093023 | 21 |
| 33 | HNF4A | 0.0112453 | 0.53080569 | 21 |
| 34 | PTGS1 | 0.00616186 | 0.50909091 | 20 |
| 35 | ARG1 | 0.01510906 | 0.52336449 | 20 |
| 36 | NOS1 | 0.00486284 | 0.53588517 | 20 |
| 37 | NR1H4 | 0.00336193 | 0.52093023 | 20 |
| 38 | HMGCR | 0.01640408 | 0.51612903 | 20 |
| 39 | NQO1 | 0.01276376 | 0.53333333 | 20 |
| 40 | LCN2 | 0.00264167 | 0.51376147 | 19 |

| **No.** | **name** | **Betweenness Centrality** | **Closeness Centrality** | **Degree** |
| --- | --- | --- | --- | --- |
| 41 | P2RY12 | 0.00547945 | 0.5045045 | 18 |
| 42 | ESR2 | 0.0015375 | 0.51851852 | 17 |
| 43 | NR1I2 | 0.0025518 | 0.51851852 | 17 |
| 44 | CYP1A2 | 0.00368791 | 0.50678733 | 17 |
| 45 | GOT2 | 0.01733753 | 0.51612903 | 17 |
| 46 | CASR | 0.00300977 | 0.5 | 17 |
| 47 | EPRS | 0.01888983 | 0.50909091 | 17 |
| 48 | ALOX15 | 0.00246129 | 0.49339207 | 16 |
| 49 | TRPV1 | 7.45E-04 | 0.51141553 | 16 |
| 50 | GPER1 | 0.00218326 | 0.5 | 16 |
| 51 | PTPN1 | 8.59E-04 | 0.51141553 | 15 |
| 52 | ITGB3 | 0.01941958 | 0.49557522 | 15 |
| 53 | CNR2 | 0.0019097 | 0.50224215 | 15 |
| 54 | CTSB | 0.00109914 | 0.5045045 | 15 |
| 55 | DPP4 | 0.00119175 | 0.49557522 | 14 |
| 56 | PLA2G2A | 0.00214099 | 0.48695652 | 14 |
| 57 | DNMT1 | 0.00249838 | 0.50909091 | 14 |
| 58 | LGALS3 | 2.84E-04 | 0.49557522 | 14 |
| 59 | S100B | 6.15E-04 | 0.49122807 | 13 |
| 60 | SYK | 0.00111788 | 0.48908297 | 13 |
| 61 | NFKB2 | 1.25E-04 | 0.49122807 | 13 |
| 62 | PRKCB | 0.00181812 | 0.49122807 | 13 |
| 63 | CBS | 0.01847281 | 0.5045045 | 13 |
| 64 | CP | 7.89E-04 | 0.50224215 | 12 |
| 65 | NR3C2 | 0.0019427 | 0.49557522 | 12 |
| 66 | CAMP | 7.32E-04 | 0.48484848 | 12 |
| 67 | CYP2C8 | 4.88E-04 | 0.46280992 | 12 |
| 68 | ITGB2 | 9.70E-04 | 0.45714286 | 12 |
| 69 | MIF | 1.88E-04 | 0.50678733 | 11 |
| 70 | HCAR2 | 4.17E-04 | 0.47659574 | 11 |
| 71 | DCN | 0.00762776 | 0.48695652 | 11 |
| 72 | GCK | 4.02E-04 | 0.47863248 | 10 |
| 73 | CES1 | 8.40E-04 | 0.47457627 | 10 |
| 74 | XDH | 0.02055604 | 0.47659574 | 10 |
| 75 | VLDLR | 0.00302109 | 0.4806867 | 10 |
| 76 | LTB4R | 5.60E-04 | 0.46090535 | 10 |
| 77 | MTTP | 0.00273499 | 0.47659574 | 10 |
| 78 | NPPB | 2.28E-04 | 0.48484848 | 10 |
| 79 | NR0B1 | 0.00158392 | 0.46090535 | 9 |
| 80 | PTK2B | 4.35E-04 | 0.48484848 | 9 |

| **No.** | **name** | **Betweenness Centrality** | **Closeness Centrality** | **Degree** |
| --- | --- | --- | --- | --- |
| 81 | CYP2D6 | 3.69E-04 | 0.46280992 | 9 |
| 82 | LYZ | 1.52E-04 | 0.47058824 | 9 |
| 83 | FURIN | 8.23E-05 | 0.48275862 | 9 |
| 84 | SUCNR1 | 1.11E-04 | 0.4375 | 9 |
| 85 | HSD11B1 | 4.78E-04 | 0.46280992 | 8 |
| 86 | HNF1A | 4.63E-04 | 0.46090535 | 8 |
| 87 | LTB4R2 | 2.61E-04 | 0.448 | 8 |
| 88 | SHBG | 1.90E-04 | 0.4806867 | 8 |
| 89 | PTGIS | 6.89E-04 | 0.44094488 | 7 |
| 90 | PIK3CG | 1.12E-04 | 0.46666667 | 7 |
| 91 | NR1H3 | 9.91E-04 | 0.44621514 | 7 |
| 92 | PPARD | 5.96E-04 | 0.47058824 | 7 |
| 93 | APRT | 0.01872156 | 0.39298246 | 6 |
| 94 | FADS2 | 2.42E-04 | 0.448 | 6 |
| 95 | HSPA2 | 1.60E-04 | 0.41791045 | 5 |
| 96 | PPP3CA | 2.31E-05 | 0.4375 | 5 |
| 97 | SOAT1 | 9.25E-04 | 0.4375 | 5 |
| 98 | SFTPD | 2.30E-05 | 0.43243243 | 5 |
| 99 | ALDH2 | 4.69E-05 | 0.4028777 | 4 |
| 100 | CAD | 1.74E-04 | 0.40727273 | 4 |
| 101 | NR1H2 | 2.43E-05 | 0.37458194 | 4 |
| 102 | SOAT2 | 1.42E-04 | 0.36963696 | 4 |
| 103 | PAEP | 0 | 0.45528455 | 4 |
| 104 | PTGIR | 0 | 0.4057971 | 4 |
| 105 | PNP | 0.00168819 | 0.33234421 | 3 |
| 106 | HDAC9 | 0 | 0.3943662 | 3 |
| 107 | PIN1 | 1.34E-05 | 0.40875912 | 3 |
| 108 | ESRRB | 3.86E-05 | 0.3943662 | 3 |
| 109 | EARS2 | 2.71E-05 | 0.39857651 | 2 |
| 110 | MTAP | 0 | 0.2835443 | 2 |
| 111 | HAL | 2.46E-05 | 0.34890966 | 2 |
| 112 | PVR | 0 | 0.33234421 | 1 |
| 113 | MTRR | 0 | 0.33633634 | 1 |
| 114 | SUCLA2 | 0 | 0 | 0 |
