# Supplementary material for: Investigating the mechanisms of Modified Xiaoyaosan (tiaogan-liqi prescription) in suppressing the progression of atherosclerosis, by means of integrative pharmacology and experimental validation
Source: Aging (Albany NY). 2021 Apr 4;13(8):11411–32. doi: 10.18632/aging.202832 (PMC8109114; doi:10.18632/aging.202832)
Supplement: Supplementary Table 5 [file aging-13-202832-s005.docx]

Supplementary Table 5. Detailed information about therapeutic effects, pharmacological actions of tern herbs contained in TGLQ and the corresponding pathways involved by TGLQ putative targets.

| Herbs | Roles of Herbs | Therapeutic effects | Pharmacological actions | Corresponding pathways |
| --- | --- | --- | --- | --- |
| Bupleurum Chinense DC.(Chaihu, CH) | Sovereign herb | ①Soothing the liver ②Regulating qi stagnation ③Replenishing qi ④Clearing heat | ①Sedative effect  ②Antipyretic and analgesic effect  ③Antioxidation effect  ④Anti-inflammatory effect  ⑤Hypotensive effect  ⑥Anticoagulant effect  ⑦Protecting liver function  ⑧Improving glucose and lipid metabolism  ⑨Protecting stomach function  ⑩Improving immune system function  ⑪Neuroendocrine regulation | ①GABA A receptor activation  ②Nuclear Receptor transcription pathway  ③SUMOylation of intracellular receptors  ④Endogenous sterols  ⑤Recycling of bile acids and salts  ⑥PPARA activates gene expression  ⑦Transcriptional regulation of white adipocyte differentiation  ⑧Synthesis of bile acids and bile salts  ⑨Synthesis of Prostaglandins (PG) and Thromboxanes (TX)  ⑩Interleukin-1 processing  ⑪HSP90 chaperone cycle for steroid hormone receptors (SHR) |
| Ligusticum chuanxiong Hort. (Chuanxiong, CX) | Sovereign herb | ①Activating blood circulation ②Regulating qi stagnation ③Dispelling the wind pathogen ④Relieving pain | ①Sedative effect  ②Dilating coronary artery  ③Improving myocardial metabolism  ④Anti-platelet aggregation effect  ⑤Anticoagulant effect  ⑥Antispasmodic and analgesic effect  ⑦Bacteriostasis  ⑧Antiatherogenic effect | ①Nuclear Receptor transcription pathway  ②SUMOylation of intracellular receptors  ③Biosynthesis of maresin-like SPMs  ④Regulation of insulin secretion  ⑤Synthesis of Prostaglandins (PG) and Thromboxanes (TX) ⑥Adrenaline,noradrenaline inhibits insulin secretion ⑦CYP2E1 reactions  ⑧Retinoid metabolism and transport |
| Curcuma wenyujin Y.H.Chen et C.Ling (Yujin, YJ) | Ministerial herb | ①Activating blood circulation ②Regulating qi stagnation ③Cooling blood ④Curing jaundice  ⑤Relieving pain | ①Protecting liver function  ②Improving immune system function  ③Bacteriostasis  ④Improving lipid metabolism  ⑤Promoting bile acid synthesis and secretion  ⑥Promoting insulin secretion  ⑦Promoting gastric acid secretion  ⑧Anti-platelet aggregation effect  ⑨Anticoagulant effect | ①Nuclear Receptor transcription pathway  ②SUMOylation of intracellular receptors  ③Synthesis of bile acids and bile salts  ④TRP channels  ⑤Transcriptional regulation of white adipocyte differentiation ⑥PPARA activates gene expression |
| Angelica sinensis (Oliv.)Diels (Danggui, DG) | Ministerial herb | ①Activating blood circulation ②Tonifying blood ③Moistening the intestines and relaxing the bowels ④Relieving pain ⑤Regulating menstruation | ①Antispasmodic and analgesic effect  ②Dilating coronary artery and improving myocardial ischemia  ③Antiarrhythmic effect ④Anti-inflammatory effect  ⑤Improving lipid metabolism  ⑥Anti-platelet aggregation effect  ⑦Anticoagulant effect  ⑧Promoting hematopoietic function  ⑨Improving immune system function  ⑩Protecting liver function  ⑪Antioxidation effect | ①GABA A receptor activation ②Citric acid cycle (TCA cycle) ③Retinoid metabolism and transport  ④Nuclear Receptor transcription pathway  ⑤SUMOylation of intracellular receptors  ⑥Synthesis of Prostaglandins (PG) and Thromboxanes (TX) ⑦Synthesis of bile acids and bile salts  ⑧PI5P, PP2A and IER3 Regulate PI3K/AKT Signaling |

**Table 2. (continued)**

| Herbs | Roles of Herbs | Therapeutic effects | Pharmacological actions | Corresponding pathways |
| --- | --- | --- | --- | --- |
| Paeonia lactiflora Pall.（Baishao, BS） | Ministerial herb | ①Softening the liver ②Tonifying blood ③Retaining yin ④Relieving pain ⑤Regulating menstruation | ①Antispasmodic and analgesic effect  ②Anti-inflammatory and anti-ulcer effect  ③Improving immune system function  ④Dilateing blood vessels  ⑤Anti-platelet aggregation effec  ⑥Bacteriostasis  ⑦Promoting hematopoietic function  ⑧Protecting liver function  ⑨Sedative effect | ①Nuclear Receptor transcription pathway  ②SUMOylation of intracellular receptors  ③Recycling of bile acids and salts  ④Synthesis of bile acids and bile salts  ⑤Interleukin-4 and Interleukin-13 signaling ⑥HSP90 chaperone cycle for steroid hormone receptors (SHR) ⑦PPARA activates gene expression ⑧Transcriptional regulation of white adipocyte differentiation ⑨Glucocorticoid biosynthesis ⑩ESR-mediated signaling ⑪FOXO-mediated transcription of oxidative stress, metabolic and neuronal genes  ⑫VEGFA-VEGFR2 Pathway |
| Atractylodes macrocephala Koidz. (Baizhu,  BZ) | Adjuvant herb | ①Fortifying the spleen ②Drying dampness and draining water ③Miscarriage prevention ④Replenishing qi | ①Diuretic effect  ②Hypoglycemia  ③Improving immune system function  ④Anticoagulant effect  ⑤Dilateing blood vessels  ⑥Anti-cancer  ⑦Bacteriostasis  ⑧Promoting hematopoietic function  ⑨Protecting liver function | ①GABA A receptor activation ②Nuclear Receptor transcription pathway  ③SUMOylation of intracellular receptors  ④Interleukin-1 processing  ⑤Synthesis of bile acids and bile salts  ⑥Interleukin-4 and Interleukin-13 signaling ⑦Transcriptional regulation of white adipocyte differentiation ⑧Activation of gene expression by SREBF (SREBP) ⑨Interleukin-10 signaling ⑩PPARA activates gene expression ⑪HSP90 chaperone cycle for steroid hormone receptors (SHR) |
| Poria cocos(Schw.)Wolf (Fuling, FL) | Adjuvant herb | ①Fortifying the spleen ②Drying dampness and draining water ③Calming the heart ④Replenishing qi | ①Diuretic effect  ②Bacteriostasis  ③Protecting liver function  ④Anti-cancer  ⑤Improving immune system function  ⑥Sedative effect | ①Nuclear Receptor transcription pathway  ②GABA A receptor activation ③SUMOylation of intracellular receptors  ④PPARA activates gene expression  ⑤RORA activates gene expression ⑥Unblocking of NMDA receptors, glutamate binding and activation  ⑦Negative regulation of NMDA receptor-mediated neuronal transmission  ⑧Long-term potentiation ⑨Activation of gene expression by SREBF (SREBP) ⑩Synthesis of bile acids and bile salts  ⑪Recycling of bile acids and salts |

**Table 2. (continued)**

| Herbs | Roles of Herbs | Therapeutic effects | Pharmacological actions | Corresponding pathways |
| --- | --- | --- | --- | --- |
| Glycyrrhiza uralensis Fisch. (Gancao, GC) | Adjuvant herb | ①Fortifying the spleen ②Clearing heat ③Resolving phlegm and suppressing cough ④Relieving pain ⑤Moderating the property of herbs ⑥Replenishing qi ⑦Detoxicating | ①Anti-digestive system ulcer  ②Promoting gastric acid secretion  ③Protecting liver function  ④Promoting bile acid synthesis and secretion  ⑤Antiarrhythmic effect  ⑥Improving lipid metabolism  ⑦Antiatherogenic effect  ⑧Eliminating phlegm and antitussive effect ⑨Anti-inflammatory effect  ⑩Sedative effect  ⑪Improving immune system function  ⑫Detoxicating effect | ①Nuclear Receptor transcription pathway  ②GABA A receptor activation ③SUMOylation of intracellular receptors  ④TP53 Regulates Metabolic Genes ⑤Interleukin-4 and Interleukin-13 signaling ⑥Synthesis of bile acids and bile salts  ⑦Transcriptional regulation of white adipocyte differentiation  ⑧Synthesis of Prostaglandins (PG) and Thromboxanes (TX)  ⑨Recycling of bile acids and salts  ⑩HSP90 chaperone cycle for steroid hormone receptors (SHR) ⑪IkBA variant leads to EDA-ID ⑫PPARA activates gene expression  ⑬Activation of gene expression by SREBF (SREBP) ⑭Glucocorticoid biosynthesis  ⑮TRAF6 mediated NF-kB activation |
| Mentha haplocalyx Briq.（Bohe, BH) | Messenger herb | ①Soothing the liver ②Regulating qi stagnation ③Dispelling the wind pathogen ④Relieving sore throat ⑤Detoxicating ⑥Clearing heat | ①Anti-virus effect  ②Analgesic and antipruritic effect  ③Eliminating phlegm and antitussive effect  ④Bacteriostasis  ⑤Promoting bile acid synthesis and secretion  ⑥Protecting liver function | ①Amino acid synthesis and interconversion (transamination) ②Mitochondrial tRNA aminoacylation ③Unblocking of NMDA receptors, glutamate binding and activation  ④TP53 Regulates Metabolic Genes  ⑤Purine ribonucleoside monophosphate biosynthesis ⑥Activation of AMPA receptors ⑦Long-term potentiation ⑧Neurotransmitter receptors and postsynaptic signal transmission |
| Zingiber officinale Rosc. (Shengjiang, SJ) | Messenger herb | ①Releasing the exterior and dissipating cold  ②Warming the middle and relieving vomitting ③Detoxicating ④Resolving phlegm and suppressing cough | ①Protecting stomach function and antiemetic effect  ②Hypertensive effect  ③Bacteriostasis  ④Antioxidation effect  ⑤Improving glucose and lipid metabolism  ⑥Anti-inflammatory effect  ⑦Anti-platelet aggregation effect  ⑧Antiatherogenic effect | ①GABA A receptor activation ②Nuclear Receptor transcription pathway  ③SUMOylation of intracellular receptors  ④TRP channels ⑤Adrenaline,noradrenaline inhibits insulin secretion ⑥Regulation of insulin secretion ⑦Synthesis of bile acids and bile salts  ⑧Recycling of bile acids and salts  ⑨Retinoid metabolism and transport |
